# Efficient CRISPR editing with a hypercompact Cas12f1 and engineered guide RNAs delivered by adeno-associated virus

**DOI:** 10.1038/s41587-021-01009-z

**Published:** 2021-09-02

**Authors:** Do Yon Kim, Jeong Mi Lee, Su Bin Moon, Hyun Jung Chin, Seyeon Park, Youjung Lim, Daesik Kim, Taeyoung Koo, Jeong-Heon Ko, Yong-Sam Kim

**Affiliations:** 1 Genome Editing Research Center, Korea Research Institute of Bioscience & BioTechnology, Daejeon, Republic of Korea; 2 KRIBB School of Bioscience, Korea University of Science and Technology, Daejeon, Republic of Korea; 3 GenKOre, Daejeon, Republic of Korea; 4 Department of Biomedical and Pharmaceutical Sciences, Graduate School, Kyung Hee University, Seoul, Republic of Korea; 5 Department of Pharmaceutical Science, College of Pharmacy, Kyung Hee University, Seoul, Republic of Korea

**Keywords:** Targeted gene repair, CRISPR-Cas9 genome editing

## Abstract

Gene therapy would benefit from a miniature CRISPR system that fits into the small adeno-associated virus (AAV) genome and has high cleavage activity and specificity in eukaryotic cells. One of the most compact CRISPR-associated nucleases yet discovered is the archaeal Un1Cas12f1. However, Un1Cas12f1 and its variants have very low activity in eukaryotic cells. In the present study, we redesigned the natural guide RNA of Un1Cas12f1 at five sites: the 5′ terminus of the *trans*-activating CRISPR RNA (tracrRNA), the tracrRNA–crRNA complementary region, a penta(uridinylate) sequence, the 3′ terminus of the crRNA and a disordered stem 2 region in the tracrRNA. These optimizations synergistically increased the average indel frequency by 867-fold. The optimized Un1Cas12f1 system enabled efficient, specific genome editing in human cells when delivered by plasmid vectors, PCR amplicons and AAV. As Un1Cas12f1 cleaves outside the protospacer, it can be used to create large deletions efficiently. The engineered Un1Cas12f1 system showed efficiency comparable to that of SpCas9 and specificity similar to that of AsCas12a.

## Abstract (web summary)

A miniature CRISPR system may enable genome editing using single AAV vectors.

## Main

The clustered regularly interspaced short palindromic repeats (CRISPR) system, which functions as an adaptive immune system in bacteria, archaea and huge bacteriophages, has been developed into versatile genome-editing tools^1–4^. Conventional genome-editing tools induce double-stranded DNA (dsDNA) breaks (DSBs), which frequently result in indel mutations through the nonhomologous end-joining (NHEJ)-mediated repair process^5,6^. Precise genetic modifications have also been achieved by homology-driven repair^7^, base editing systems^8,9^ and prime editing technology^10^. These diverse genome-editing tools have facilitated cell engineering, generation of model animals, development of new plant varieties^11^ and genetic screening^12^. In particular, these methods hold promise for gene therapy in the treatment of cancer^13^, genetic disorders^14^ and infectious diseases^15,16^.

Gene therapy can be performed in vivo or ex vivo, depending on the accessibility of target cells. Ex vivo therapy is performed on individual types of patient-derived cells, which include immune cells^17^, adult stem cells^18^ or, in principle, germ cells^19^. However, most monogenic diseases require systemic delivery of genetic materials through an in vivo strategy, which necessitates an efficient vehicle for their delivery. AAV is a US Food and Drug Administration-approved vehicle as a result of its safety, persistence and compatibility with mass production^20^. In this regard, an AAV-loadable CRISPR tool would hold promise for the treatment of genetic disorders in vivo^21^. However, AAV has a limited payload size of <4.7 kb which hampers clinical applications of most CRISPR tools^22^. Smaller Cas proteins, including SaCas9 (ref. ^23^) and CjCas9 (ref. ^24^), have been identified and validated with respect to their use as genome-editing tools delivered through an AAV. These can be delivered with an AAV, but even smaller CRISPR systems are needed to enable AAV delivery of base editors^8,9^, prime editors^10^ or regulators of epigenetic features^5^. Moreover, a miniature CRISPR system would allow the use of multiple guide (g)RNAs and/or additional regulatory genes in a single AAV particle.

Classified as type-V CRISPR nucleases, miniature Cas proteins, including Cas12f (also known as Cas14) and Cas12j (CasΦ), were identified from archaea^25^ and huge bacteriophages^2^. Miniature CRISPR–Cas effectors consist of ~400–700 amino acid residues and include a single RuvC nuclease domain^25^. So far, it has not been demonstrated that miniature Cas proteins can be used as robust genome-editing tools in eukaryotic cells. Cas12f1 was originally reported to show only single-stranded DNA (ssDNA) cleavage activity^25^. A subsequent study revealed indel activity of Cas12f in eukaryotic cells, but the efficiency was only marginal and only two targets were tested^26^. Cas12j was functionally validated in plant cells, but the indel efficiency was also <1%^2^. Furthermore, in neither case was the feasibility of AAV delivery demonstrated.

In the present study, we intensively remodeled the gRNA for Un1Cas12f1 (hereafter referred to as Cas12f) through five rounds of gRNA engineering. The individual changes to the gRNA structure were synergistic, thereby transforming the CRISPR–Cas12f system into a highly efficient and specific genome-editing tool. Moreover, the gRNA remodeling substantially reduced the gRNA size. When delivered by an AAV vector, this compact CRISPR system enabled multiplexed and efficient genome editing. In the present study, we propose that our engineered Cas12f1 system is useful for gene editing with AAV delivery and that Un1Cas12f1 might form the basis for developing AVV-deliverable base and prime editors, epigenetic regulators and constructs for site-specific gene regulation.

## Results

### Overall strategy for gRNA engineering

**Fig. 1:**
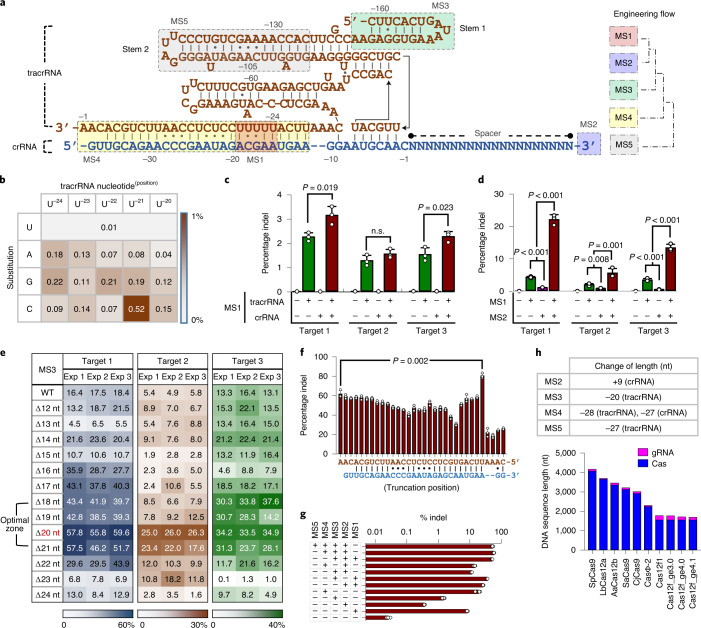
Engineering Cas12f gRNA. **a**, Structure of the canonical Cas12f1 gRNA consisting of tracrRNA and crRNA. Five MSs for gRNA engineering are indicated. The gRNA engineering steps were performed sequentially, from MS1 to MS2 and MS3, and finally MS4; MS5 modifications are discussed elsewhere. **b**, Increased Cas12f-mediated indel frequencies caused by substitutions of uridine in the tracrRNA penta(uridinylate) site (MS1). **c**, Combined effects of sequence modifications in both the tracrRNA and the crRNA at the penta(uridinylate) site on indel frequencies (*n* = 3). **d**, Synergistic modulation of indel frequencies by modifications in MS1 and MS2 (addition of poly(uridinylate) 3′ overhang on the crRNA) (*n* = 3). **e**, Optimal length of truncation of the 5′ terminus of the tracrRNA (MS3). Values were obtained from independent triplicate experiments. **f**, Changes in indel frequencies induced by the truncation of the crRNA–tracrRNA complementary region (MS4). At each position, the crRNA and tracrRNA were truncated and connected with a GAAA tetraloop (*n* = 3). **g**, Increased indel frequencies induced by various combinations of gRNA modifications (*n* = 3). **h**, Changes in the length of the crRNA and tracrRNA caused by each step of gRNA engineering and comparison of the lengths of sequences encoding the components of several representative CRISPR–Cas systems. NS, not significant. **c**,**d**,**f**, Two-group and multiple comparisons were performed by the two-sided Student’s *t*-test and one-way ANOVA test, respectively. All error bars represent s.d. Source data

Efforts to engineer gRNAs have contributed to improving CRISPR tools with respect to efficiency of indel generation^27^, simplicity^4^, multiplexing^28^, imaging^29^ and specificity^4,30^. To optimize the gRNA of Cas12f, we used knowledge from past gRNA engineering efforts and identified five potential modification sites (MSs) throughout the tracrRNA and crRNA sequences as follows: MS1, an internal penta(uridinylate) (UUUUU) sequence in the tracrRNA; MS2, the 3′ terminus of the crRNA; MS3, the ‘stem 1’ region of the tracrRNA; MS4, the tracrRNA–crRNA complementary region; and MS5, the ‘stem 2’ region of the tracrRNA. The gRNA engineering steps proceeded sequentially from MS1 to MS5 (Fig. 1a). As the thermodynamically unfavorable base-paring of the natural tracrRNA and crRNA (Supplementary Fig. 1a) would nullify the effects of gRNA engineering, we used a single-guide RNA (sgRNA) connected with a GAAA loop as a precursor for the series of gRNA engineering steps.

#### MS1: correcting an internal penta(uridinylate) sequence

The canonical Cas12f1 tracrRNA contained an internal UUUUU sequence that spanned positions −24 to −20 (numbered 5′ to 3′), as reported previously^25^. The five consecutive thymidinylates in a template would prevent the production of a full-length tracrRNA under the H1 and U6 promoters^31^. Therefore, we designated the penta(uridinylate) site (MS1) as the starting point for gRNA engineering.

To remove the termination cue, we replaced each U with a non-U nucleotide and investigated the indel frequencies at an endogenous target (target 1) in HEK293T cells (hereafter, please refer to Supplementary Table 1 for target information). Deep-sequencing analyses revealed that each substitution yielded at least a fourfold increase in indel frequency with a much higher increase (about 50-fold) by the substitution of U^−21^ with C (Fig. 1b). For further gain, we compared possible combinations for the penta(uridinylate) site by fixing the C substitution at the U^−21^ position, and substituting other uridines with the nucleotides, resulting in the highest indel frequencies, as shown in Fig. 1b. Comparative analysis revealed that substituting UUUUU with 5′-GUGCU in the tracrRNA further increased the efficiency of indel generation (Supplementary Fig. 1b). A similar screening and comparative analysis found that 5′-AGCAA in the crRNA was an optimal counterpart for the 5′-GUGCU in the tracrRNA (Supplementary Figs. 1c–e). The substitutions in the crRNA (that is, 5′-AGCAA) alone did not increase the indel frequency, but significantly improved indel efficiencies were achieved with the concomitant modification of crRNA and tracrRNA at MS1 (Fig. 1c).

#### MS2: adding 3′-poly(uridinylates) to the crRNA

Previously, we reported that a poly(uridinylated) (U-rich) 3′ overhang on the crRNA increased Cas12a-mediated indel frequencies, making them comparable to those of SpCas9 (ref. ^32^). As Cas12f1 shows a similar domain architecture to Cas12a, we explored whether a similar U-rich crRNA modification (MS2) would affect Cas12f1-mediated indel frequencies. In line with our previous results, the addition of Ts stimulated Cas12f1 activity until a T_5_ or T_6_ termination sequence was generated (Supplementary Fig. 2a). An adenylate (A) was incorporated after TTTT to obviate the termination signal for the U6 promoter, and the number of uridines in the crRNA was further increased by adding thymidinylates next to the adenylate. When a 5′-TTTTATTTTTT sequence was added to the 3′ terminus of the crRNA to create a U-rich 3′ overhang, indel frequencies were maximized. We changed the intervening A into C or G and found that a 5′-U_4_RU_4_ (R = A or G) was an optimal overhang in that position (Supplementary Fig. 2b; please refer to our previous report^32^ for detailed information). When combined in an sgRNA, the MS1 and MS2 modifications showed synergistic effects, yielding significant increases in indel frequencies, by up to 1,148-fold for target 1 (Fig. 1d). In contrast, we observed only marginal levels of indel frequencies with a canonical gRNA (percentage indels ≤0.1%). The MS1-/MS2-modified sgRNA was then subjected to further rounds of gRNA engineering.

#### MS3: truncating the 5′ region of the tracrRNA

Cas12f1 has an exceptionally long gRNA due to an oversized tracrRNA^25^. We hypothesized that the entire tracrRNA is unlikely to participate in interactions with the compact Cas12f1. A recent study also indicated that the stem 1 region is in a structurally disordered state^33,34^. To test this hypothesis, we either extended or trimmed the tracrRNA in the stem 1 region. A tracrRNA with an 18-nt truncation caused a significant increase in indel frequency (Supplementary Fig. 3a). To determine the optimal truncation, we tested a set of tracrRNA truncations beginning at positions −149 to −137. The results confirmed that a 5′ truncation of 18–21 nt yielded a highly potent sgRNA (Fig. 1e) and used a tracrRNA with a 20-nt truncation as a basis for additional engineering steps.

#### MS4: truncating the tracrRNA–crRNA complementary sequence

We sought to further trim the sgRNA without compromising indel frequencies at the crRNA–tracrRNA complementary region. We generated sgRNAs with different lengths of the tracrRNA–crRNA complementary region (Supplementary Fig. 3b). An elongated crRNA was suggested to improve the function of the CRISPR–Cas12a system^35^; thus, we tested an elongated sgRNA (+13 bp) in addition to four shortened sgRNAs. The elongated sgRNA showed a marked decrease in indel-generating efficiency, but all trimmed sgRNAs retained efficiency.

To pinpoint the optimally trimmed sgRNA, we tested various truncations at 1-bp resolution. Notably, a truncation of the entire complementary sequence resulted in even higher editing efficiency (Fig. 1f). The truncated region exactly matched the previously reported disordered region^33,34^.

We tested substituting the GAAA tetraloop linking the tracrRNA and crRNA with a hammerhead ribozyme to produce an sgRNA at the expression stage, followed by generation of a dual gRNA with an overhang on either the tracrRNA or the crRNA after self-cleavage (Supplementary Fig. 3c,d). However, none of these possibilities showed increased efficiency compared with the sgRNA with a total of 59-nt truncation.

#### MS5: truncating the stem 2 region in the tracrRNA

We considered one more possible modification site, the tracrRNA stem 2 region, because the segment spanning from A^−129^ to U^−103^ was also reported to be disordered^33,34^. Thus, we also trimmed this region at 1-bp resolution, keeping the 5′-UUAG loop preserved. Though the modified gRNAs did not further enhance the efficiency achieved by MS2/MS3/MS4 gRNA at target 2, deletion from C^−131^ to G^−101^ increased indel frequencies by about 2.7-fold at a *GAK* locus (Supplementary Fig. 3e). Validations using more targets revealed that the 27-nt truncation at the stem 2 region mediated increased indel frequencies, particularly for targets with relatively low indel frequencies generated by gRNA MS2/MS3/MS4 (Supplementary Fig. 3f). The truncation of the stem 4 region nullified the MS1–MS5 engineering (Supplementary Fig. 3g), and was not included for final engineering. Various combinations of MS modifications yielded different increases in indel frequencies (Fig. 1g). When MS1/MS2/MS3, MS2/MS3/MS4 or MS2/MS3/MS4/MS5 modifications are combined, the engineered Cas12f1 systems showed the highest genome-editing performance and thus are referred to as Cas12f_ge3.0, Cas12f_ge4.0 and Cas12f_ge4.1, respectively. It is noteworthy that the MS1 site was removed during MS4 engineering and, therefore, MS1 and MS4 engineering are mutually exclusive. The Cas12f1_ge4.1 system is characterized by highly efficient, very compact genome editors, with gRNA down-sized by almost 40% (Fig. 1h). Taken together, our extensive gRNA engineering efforts yielded a potent, extremely compact CRISPR–Cas12f1 system.

Finally, we sought to explain how each gRNA modification (MS1–MS5) contributes to increased indel frequencies using targeted RNA-sequencing (RNA-seq) analysis (Supplementary Fig. 4a). As expected, the MS1 engineering led to a drastic increase in the expression of the full-length sgRNA (Supplementary Fig. 4b,c). Besides increasing the affinity of the Cas–gRNA interaction as suggested previously^32^, the U-rich 3′ overhang appeared to stabilize the sgRNA transcript in cells. The MS3 engineering was not associated with changes in gRNA expression, but further increased the dsDNA cleavage activity of Cas12f1 when stacked to the MS1/2 modifications (Supplementary Fig. 4d). The MS4 and MS5 engineering further increased the cleavage activity. To validate the effects of the MS3 modification on indel frequencies, MS1/2- and MS1/2/3-modified gRNAs were compared with respect to indel-generating efficiency in vivo. Out of 19 targets tested, 17 (90%) showed increased indel frequencies, by at least twofold, with the average fold increase being 3.12 (Supplementary Fig. 4e). The structures of the Cas12f_ge3.0, Cas12f_ge4.0 and Cas12f_ge4.1 gRNAs are presented in Supplementary Fig. 5.

### Large-scale validation of Cas12f

**Fig. 2:**
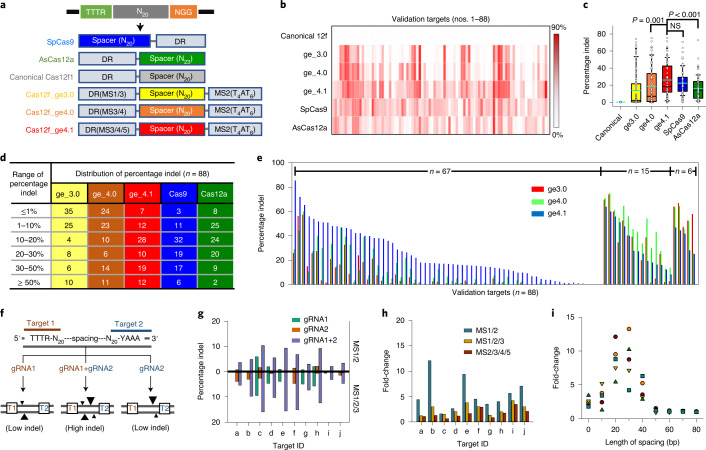
Large-scale validation of the engineered CRISPR–Cas12f system. **a**, Common sequences targetable by the SpCas9, AsCas12a and Cas12f systems and the gRNA formulations for each system. TTTR indicates TTTA or TTTG. **b**, A heatmap for indel frequencies per target obtained by SpCas9, AsCas12a or Cas12f. Measurement of indel frequencies in HEK293T cells transfected with SpCas9, AsCas12a, canonical Cas12f or engineered Cas12f vector constructs. Cells (1.75 × 10^5^) were transfected with 2 μg of plasmid vector using a Fugene lipofection kit and grown for 96 h. **c**, A box-and-whisker plot for SpCas9-, AsCas12a- and Cas12f-induced indel frequencies merged with a dot plot. Whole data points (*n* = 88) were plotted with mean values as indicated by the horizontal cyan-colored line. Box plots represent the median with interquartile ranges (25–75%); whiskers extend to 1.5× the interquartile distance from the box. *P* values were derived by a Mann–Whitney *U*-test. NS, not significant. Error bars represent the s.d. **d**, Distribution of the number of targets per indel frequency subdivision. Values indicate the number of targets with indel efficiency belonging to the indicated ranges. The indel efficiencies and target information were provided in a source file and Supplementary Table 2. **e**, Distribution of the targets that show the highest efficiencies by the _ge3.0, _ge4.0 or _ge4.1 version. **f**, Schematic representation of the paired gRNA strategy for increasing Cas12f-mediated indel frequencies. DNA cleavages are centered in the spacing region, which is 10–30 bp in length. The size of the triangle indicates the frequency of a DNA-strand cleavage. **g**, Indel frequencies induced by Cas12f with engineered gRNA at ten targets when using either or both gRNAs. The upper and lower panels indicate indel frequencies for gRNAs with MS1/2 and MS1/2/3 engineering, respectively. **h**, Comparison of fold-changes in indel frequencies caused by MS1/2- and MS1/2/3-engineered gRNAs. The fold-changes were calculated from the indel frequencies induced by paired gRNAs compared with that of a gRNA that induces a higher indel frequency at a target located between the two paired gRNAs. **i**, Fold-changes in indel efficiencies by paired gRNAs according to the length of spacing. Source data

We next investigated whether the increased genome-editing efficiency of the engineered gRNAs can be validated at a wider range of targets. We searched in silico for endogenous targets containing the sequence 5′-TTTR-N_20_-NGG-3′, which are targetable with SpCas9, AsCas12a and Cas12f1 (Fig. 2a). We randomly selected 88 such endogenous loci (for target information, please refer to Supplementary Table 2) and measured the SpCas9-, AsCas12a- and Cas12f-mediated indel frequencies in HEK293T cells. Cas12f with canonical gRNAs generated indel frequencies of <1.0% over all tested targets, with 91% (80 of 88) of targets showing frequencies of <0.1%. However, use of our engineered gRNAs led to significant increases in indel frequencies at most target sites (Fig. 2b). The average efficiency of Cas12f_ge4.1 was comparable to that of SpCas9 (*P* > 0.05) and was even higher than that of AsCas12a (Fig. 2c). The average increase in efficiency induced by Cas12f_ge4.1 sgRNA was 867-fold. Notably, Cas12f_ge4.1 had more targets with high indel frequencies (≥50%) than SpCas9 and AsCas12a (Fig. 2d). In addition, Cas12f_ge4.1 showed higher efficiencies for 76.1% (67 of 88) of targets, compared with the Cas12f_ge3.0 and Cas12f_4.0 versions, whereas the Cas12f_ge4.0 and Cas12f_ge3.0 versions were most effective for 17.0% and 6.8% of targets, respectively (Fig. 2e).

We then sought to refine the Cas12f system further, because there still remained targets resistant to genome editing by Cas12f (in fact, the situation is also true for Cas9 and Cas12a, but Cas12f1 showed more targets with indel frequencies <1% than SpCas9). We hypothesized that the low efficiency of Cas12f1 at certain sites may originate from different cleavage efficiency between target and nontarget strands, because the compact size of Cas12f might cause less efficient nontarget strand cleavage. To test this hypothesis, we selected targets that carry a 5′-TTTR-N_20_-spacing-N_20_-YAAA-3′ sequence, where ‘spacing’ is a 10- to 80-bp-long dsDNA segment (Fig. 2f). These sequences are targetable by a pair of gRNAs oriented in opposite directions; two dsDNA cleavage events occur in the spacing region. Although each gRNA alone mediated relatively low indel frequencies, targets in ten loci showed sharply increased indel frequencies with the paired gRNAs. The fold increase varied among targets, but all tested targets showed indel frequencies of >1%. Moreover, final indel frequencies were further improved by using MS1/MS2/MS3- versus MS1/MS2-modified gRNAs (Fig. 2g), mainly because indel-generating efficiencies of each gRNA were increased by MS3 engineering. However, the fold increase was more pronounced for MS1/MS2 engineering, compared with the MS1/MS2/MS3 and MS2/MS3/MS4/MS5 versions (Fig. 2h). This result would be explained by our hypothesis that Cas12f1 displays unequal cleavage kinetics for the target and nontarget strands, and that the degree of difference is reduced by MS3 engineering. A longer spacing region of ≥50 bp did not yield this pair gRNA-assisted increase in indel frequencies (Fig. 2i).

### Favorable kinetic property for Cas12f-induced DNA cleavage

**Fig. 3:**
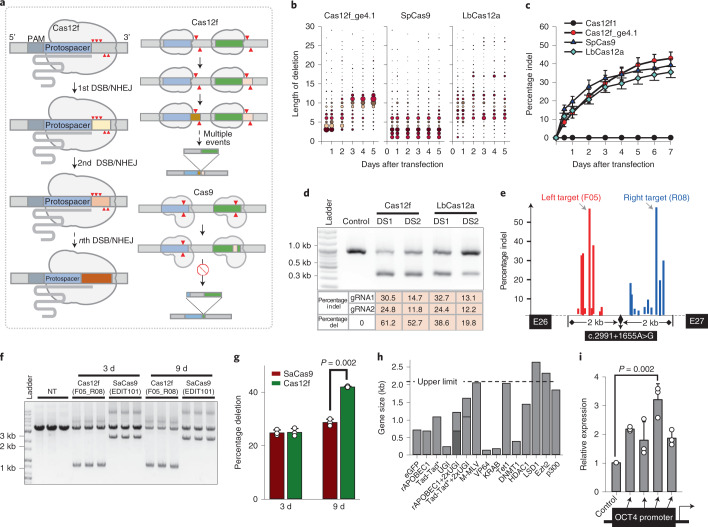
Highly efficient correction of pathogenic mutations through AAV-delivered Cas12f. **a**, A schematic illustration showing multiple chances for dsDNA cleavage and NHEJ cycles for the Cas12f system. Cleavage sites are marked with triangles and different colors indicate changes in the DNA sequences. The protospacer regions are colored sky-blue and green. **b**, Increased frequency of long deletion mutations over time in HEK293T cells transfected with CRISPR–Cas12f_ge4.1. **c**, Time-course of Cas12f-, SpCas9- and LbCas12a-induced indel frequencies in HEK293T cells transfected with plasmid vectors (*n* = 3). **d**. Comparison of Cas12f- and LbCas12a-mediated rates of exon 51 deletion from the human dystrophin gene in AC16 cells. The lower bands indicate the PCR amplicons of the exon 51-deleted locus. The intensity of the lower bands is indicative of the deletion efficiency. Deletion strategy 1 (DS1) and DS2 target identical loci for Cas12f and Cas12a. The data represent three experiments. **e**, Screening of targets for the deletion of the c.2991+1655A>G mutation from the *CEP290* gene. **f**, Comparison of Cas12f- and SaCas9 (EDIT101)-mediated frequencies of deletion of the c.2991+1655A>G mutation. HEK293T cells (2 × 10^5^) were seeded into 12-well plates, transduced with AAV2 harboring the Cas12f or SaCas9 system at 5.0 × 10^9^ vector genomes (vg) ml^−1^, and harvested 3 and 9 d post-transduction. NT, nontransduction. The data represent three experiments. **g**, Quantitative analysis of deletion frequencies using RT-qPCR. Percentage deletion indicates the percentage ratio of PCR amplicons containing the deletion versus intact amplicons (*n* = 3). *P* values were derived using a two-sided Welch’s *t*-test. **h**, Possible applications of Cas12f using an AAV delivery system. For AAV delivery of vector constructs harboring the Cas12f sequence with two nuclear localization signals, a BGH poly(A) signal, and an XTEN linker sequence under the control of an EF-1α core promoter and a ge4.1 sequence under the control of a U6 promoter, a protein encoded by a gene ≤2.1 kb in size could be fused to Cas12f. **i**, Application of dCas12f-VP64 to CRISPRa. The fusion protein guided by gRNAs (ge4.1) targeting promoter regions of *OCT4* led to transcriptional activations in HEK293T cells (*n* = 3). *P* values were derived using a two-sided Student’s *t*-test. All error bars represent the s.d. Source data

In addition to the compactness of Cas12f1, this system has an additional advantage for gene therapy: it induces dsDNA cleavages outside the protospacer sequence^33,36^. This property implies that, even after the initial round of NHEJ-mediated indel mutations, the protospacer sequence is likely to remain unchanged. Then, further rounds of the dsDNA cleavage–NHEJ process can continue (Fig. 3a). This property is even more desirable for a large DNA-deletion strategy involving a pair of gRNAs. We analyzed the profile of indel mutations induced by Cas12f. Most mutation patterns included relatively long deletions that affected the protospacer sequence (Supplementary Fig. 6a,b). In contrast, indel mutations outside the protospacer were relatively rare. We interpreted these long deletions to be the products of multiple cutting-and-joining processes. In fact, this assumption was confirmed through a time-course investigation of indel patterns. In the early phase of transfection, deletions of <5 bp were dominant (Fig. 3b; the radius of a bubble indicates the mutation frequency). However, the frequency of long deletions increased over time until 4 d later. In contrast, the pattern of indel mutations was almost consistent over time for SpCas9 and LbCas12a. Moreover, Cas12f caused a more persistent increase in indel frequencies, compared with SpCas9 and LbCas12a (Fig. 3c).

A handful of genetic disorders can potentially be treated by deletion of pathogenic introns or exons using paired gRNAs and Cas proteins, including Duchenne muscular dystrophy^37^, Leber congenital amaurosis 10 (LCA10)^38^ and Usher’s syndrome type 2A^39^. We explored the potential utility of the Cas12f system for those applications. As a case study, we selected a pair of sites in the vicinity of exon 51 of the human dystrophin gene that are common targets for LbCas12a and Cas12f. Screening experiments identified target sequences that show similar indel frequencies for LbCas12a and Cas12f. Despite the similar indel efficiencies of individual gRNAs, Cas12f resulted in a higher level of deletions, compared with LbCas12a (Fig. 3d). These results indicate that Cas12f might be particularly useful for AAV delivery in gene therapy applications that require deletions.

### AAV delivery of the engineered Cas12f system

Next, we investigated the genome-editing performance of a recombinant AAV2 (rAAV2)–Cas12f vector. We constructed an rAAV vector carrying sequences encoding either Cas12f_ge4.1 or a control vector (scrambled sgRNAs). Cas12f1 and sgRNA expression were driven under the control of the chicken β-actin and the human U6 promoters, respectively (Supplementary Fig. 7a). The total length of these sequences (4.40 kb) fell within the permissive size for an AAV payload, even in the presence of two sgRNA sequences and an enhanced green fluorescent protein (eGFP)-encoding reporter sequence. The rAAV2 particles were produced in HEK293T cells after transfection with an rAAV vector, pAAVED2/2 and a helper plasmid. The sgRNAs respectively targeted an intergenic locus (target 1) and the *KRT1* gene (target 2).

AAV delivery to HEK293T cells led to an increase of the frequencies of indel mutations over time (Supplementary Fig. 7b) and with increasing numbers of rAAV2 particles (Supplementary Fig. 7c). The infection was monitored by green fluorescence, which was persistent for 2 weeks post-transduction (Supplementary Fig. 7d).

Next, we explored the targeting of therapeutically useful loci for the deletion of a pathogenic cryptic exon in the *CEP290* gene for the treatment of LCA10 (ref. ^38^). We tested on both sides of the c.2991+1655A>G mutation site and identified a pair of highly potent sgRNAs (Fig. 3e). We then constructed an rAAV vector carrying the Cas12f1_ge4.1 system, and the deletion-inducing efficiency of Cas12f was compared with that of SaCas9 (EDIT101, a gene therapeutic agent under clinical trial) in HEK293T cells. We observed higher levels of deletions on agarose gels for the Cas12f system (Fig. 3f). Quantitative analysis using droplet digital PCR showed a 46% higher deletion rate of Cas12f, compared with EDIT101 (Fig. 3g). These results indicate that Cas12f might provide a versatile and valid genome-editing platform for gene therapy.

When using an elongation factor (EF)-1α core promoter, a bovine growth hormone (BGH) poly(A) signal sequence, a U6 promoter and an XTEN linker between sequences encoding Cas12f1 and a potential fusion partner are used, we have an upper limit of approximately 2.1 kb for a fusion partner gene for AAV delivery. Considering the sizes of genes encoding validated regulators, we propose that the Cas12f system could provide a scaffold for various applications including CRISPR interference (CRISPRi)^40^, CRISPR activation (CRISPRa), base editing^8,9^, prime editing^10^ and site-specific epigenetic regulations^5,6^ (Fig. 3h). The possibility of such applications was explored in a CRISPRa strategy, where dCas12 (D510A) fused to VP64 activated transcriptional expression of *OCT4* gene in a gRNA-dependent manner (Fig. 3i).

### Genome-editing specificities of Cas12f

**Fig. 4:**
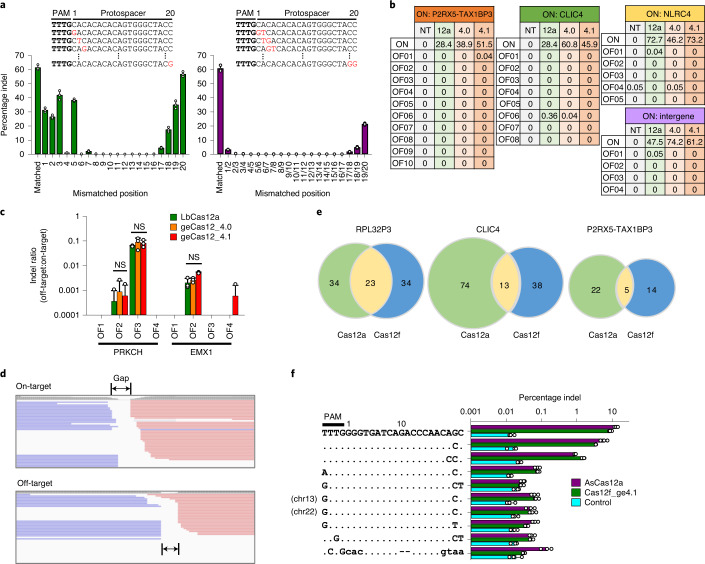
Unbiased and targeted analysis of Cas12f specificity as assessed by Digenome-seq analysis. **a**, Tolerance of Cas12f_4.1 to mismatched gRNA. The engineered gRNAs with a singly mismatched base and pairs of mismatched bases were used for the investigation of indel frequencies in HEK293T cells (*n* = 3). **b**, Indel frequencies at off-targets identified by OFFinder for AsCas12a, Cas12_ge4.0 and Cas12_ge4.1. An intergene corresponds to target 3 targeted throughout the main text. **c**, Indel frequencies at previously validated off-targets for AsCas12a, Cas12_ge4.0 and Cas12_ge4.1. The ratio of indel frequency at off-target to that at on-target was considered as an index for specificity (*n* = 3). Statistical analysis was performed by a two-sided Student’s *t*-test. NS, not significant. **d**, IGV files at on-target and off-target loci after in vitro digestion of genomic DNA. The gap indicates a region where sequences were missing in both forward and reverse reads. **e**, The number of potential off-target loci identified by Digenome-seq analysis for AsCas12a and Cas12f. **f**, Validation of off-target sites identified by Digenome-seq analysis using Cas12f and AsCas12a as endonucleases. Indel frequencies were measured at both on-target and potential off-target loci after transfection with either Cas12f- or AsCas12a-encoding vector. Control refers to Cas12f-untreated cells (*n* = 3). All error bars represent the s.d. Source data

Considering the persistent activity of Cas12f in cells (Fig. 3c,f,g), it is particularly important to examine the specificity of this system. First, we assessed the activity of Cas12f when gRNA_ge4.1 contained single- or adjacent two-base mismatches with the protospacer complementary sequence. Certain levels of tolerance were observed for single-base mismatches, particularly at positions 1–3, 5 and 17–20 (Fig. 4a). To compare the results with that of Cas12a^32,41^, Cas12f showed lower tolerance in the protospacer-adjacent motif (PAM)-proximal regions and similar or slightly higher tolerance in the PAM-distal regions (positions 17–20). However, Cas12f exhibited less tolerance for mismatches in the middle region (positions 6–16). Moreover, Cas12f showed negligible levels of tolerance for two-base mismatches, except for positions 19/20, again similar to Cas12a.

Next, we employed targeted approaches to assess specificity. Using Cas-OFFinder^42^, we selected potential off-target sites that contained three base mismatches, but no bulges, with a set of on-target sites in *P2RX5-TAX1BP3*, *CLIC4*, *NLRC4* and an intergene region, for which Cas12f showed higher on-target efficiencies than Cas12a (Fig. 4b and Supplementary Table 3). Deep-sequencing analysis revealed that Cas12f was more specific than AsCas12a: whereas AsCas12a resulted in residual levels of indels (<0.1%) at two off-target sites and an indel frequency of 0.36% at one other site among a total of 26 potential off-target sites, Cas12f_ge4.0 and _ge4.1 resulted in an indel frequency of 0.04% at each one of the potential off-target sites. We also compared genome-editing specificity for targets in *RPL32P3*, *PRKCH* and *EMX1*, for which Cas12a was previously observed to induce off-target effects^41,43^ (Supplementary Table 3). On the whole, Cas12f and AsCas12a induced similar off-target effects, except for the off-target sites that had a single mismatch in the PAM-distal region (OF1–3 for *RPL32P3*; Fig. 4c).

We next employed the Digenome-sequencing (Digenome-seq) analysis to further examine the specificity of Cas12f^44^. Three targets (*RPL32P3*, *CLIC4* and *P2RX5-TAX1BP*3) were selected to compare the specificity of AsCas12a and Cas12f_ge4.1. Analysis of the Integrative Genomics Viewer (IGV) files from the Cas12f_ge4.1 experiments shows a presence of gaps between forward- and reverse-strand reads at both on-target and off-target sites (Fig. 4d), which is assumed to arise from either the ssDNA cleavage activity by the cleavage-activated Cas12f^25^ or a generation of 3′ overhang. The Digenome-seq analysis revealed that gRNAs targeting *RPL32P3*, *CLIC4* and *P2RX5-TAX1BP*3 showed off-target activity for Cas12f at 57, 51 and 19 loci, respectively, which were similar to or smaller in number than 57, 87 and 27, respectively, for AsCas12a (Fig. 4e and Supplementary Table 4). Intrinsically, Cas12f would be expected to show fewer off-target sites than Cas12a because of the more restricted preference of PAMs^36^. We then validated the nine potential off-target sites for *RPL32P3* by measuring Cas12f- and AsCas12a-mediated indel frequencies. The indel frequencies at the on-target site were similar for Cas12f and AsCas12a. Similarly, the indel frequencies at off-target sites were not significantly different between the two CRISPR systems, although Cas12f showed slightly higher off-target activity at the sites with a mismatch in the PAM-distal region, in line with Fig. 4a. In addition, a certain level of indel frequencies was observed for noncanonical TTTR PAM, such as GTTG and ATTG, for both enzymes. However, the overall indel frequencies at the investigated sites were similar between Cas12f and AsCas12a, indicating that Cas12f shows high genome-editing specificity comparable to Cas12a (Fig. 4f). The Cas12f system not only recognized fewer off-target sites, but also resulted in lower off-target/on-target indel frequency ratios. Despite the lower off-target activity, Cas12f showed long deletions (up to ~10 kb), as is observed for SpCas9 and AsCas12a (Supplementary Fig. 8), which requires further scrutiny^45^.

## Discussion

Cas12f1 has an extra-long gRNA for its compact protein size, which might be related to Cas12f1’s ssDNA cleavage activity. Our engineered sgRNA_ge4.1, although still a little bit longer, was structurally similar to the crRNA used by Cas12a or Cas12j. This architecture was obtained mainly by trimming the 5′-tracrRNA, crRNA–tracrRNA hybridization regions and stem 2 region. In fact, those gRNA modifications led to a substantial improvement in dsDNA cleavage activity. Namely, the MS3, MS4 and MS5 modifications are thought to improve the compatibility between the gRNA and Cas12f, whereas the MS1 modification addresses a problem arising from differences between the prokaryotic and eukaryotic expression systems. We also present evidence that MS2 modifications contribute to both aspects. It is interesting that our empirical approach to derive an optimized gRNA was in line with recent structural studies, in that the trimmed regions are structurally disordered^33,34^. One interpretation would be that a disordered region might hinder Cas12f1 homodimerization. Through extensive gRNA remodeling, we transformed Cas12f1 into an efficient genome-editing tool. Large-scale validation experiments indicated that, together with Cas9 and Cas12a, Cas12f1 provides a universal genome-editing platform. In particular, the engineered Cas12f platform could provide a versatile means of gene therapy deliverable by an AAV in clinical settings.

The utility of CRISPR technology has been enormously expanded by engineering catalytic variants of Cas proteins^46–48^ and fusion with other functional proteins. Catalytically inactive Cas proteins have been fused to transcriptional regulators, thereby achieving tailored gene expression^5^. Base modifiers fused to catalytically inactive Cas or Cas nickase^17^ enable precise genome editing at single-nucleotide resolution by minimizing dsDNA breaks^49^. In particular, prime editing systems rely on a fusion of nCas9 and M-MLV reverse transcriptase for DNA modifications^10,50^. Despite their functionalities, clinical applications of base and prime editors are limited by their being overweight beyond the limits of the genetic payload in an AAV. Compact Cas proteins, such as Cas12f, should enable the creation of precise genome-editing tools that are easier to deliver with AAVs.

Cas12f is particularly efficient in the deletion of a pathogenic exon or intron using paired gRNAs with expression individually driven by U6 or H1 promoters. Usher’s syndrome^39^, Duchenne muscular dystrophy^37^ and a certain type of LCA^38^ could potentially be treated by such a Cas12f-based strategy. To broaden the range of treatable diseases, it will be important to engineer catalytic variants of Cas12f1, which will benefit from the recent structural elucidation of Cas12f1 (refs. ^33,34^), as witnessed for SpCas9 (ref. ^51^) and Cas12a^52^.

## Methods

### Plasmid vector construction

The Cas12f1 gene underwent codon optimization for expression in human cells (Supplementary Fig. 9), and the optimized sequence was synthesized for vector construction (Bionics). The final sequence included the chicken β-actin promoter, a nuclear localization signal sequence at both the 5′ and the 3′ termini, and sequences encoding eGFP linked by a self-cleaving T2A peptide. Template DNAs encoding gRNAs were synthesized and cloned into a pTwist Amp plasmid vector (Twist Bioscience). If necessary, these vectors were used as a template for amplification of gRNA-encoding sequences using a human U6-complementary forward primer and a protospacer complementary reverse primer. For the construction of dual gRNA plasmids, oligonucleotides encoding tracrRNA and crRNA were cloned into pSilencer 2.0 (Thermo Fisher Scientific), using BamHI and HindIII restriction enzymes (New England Biolabs). The Cas12f_ge3.0, ge4.0 and _ge4.1 vectors were constructed by cloning the engineered gRNA-encoding oligonucleotides into vectors harboring the codon-optimized Cas12f gene using Gibson assembly; pSpCas9(BB)-2A-EGFP (PX458) v.2.0 (Addgene) was used as a backbone plasmid.

### Guide RNA engineering

The 3′-terminal modification of the crRNA (MS2) was performed with a reverse primer harboring a 3′-poly(uridinylate) sequence using Pfu PCR Master Mix5 (Biofact). The PCR amplicons were purified using a HiGene Gel&PCR Purification System (Biofact). Internal modifications of gRNA (MS1, MS4 and MS5) were performed by cloning synthetic oligonucleotides carrying modified sequences (Macrogen) into the gRNA-encoding vector linearized using ApoI and BamHI restriction enzymes. The 5′-terminal modification of the tracrRNA (MS3) was conducted by PCR amplifications using a forward primer targeting the 5′-tracrRNA region and a reverse primer targeting the human U6 promoter region. The gRNA-encoding sequences for each engineering step were compiled in Supplementary Table 5. PCR amplifications were performed using Q5 Hot Start high-fidelity DNA polymerase (New England Biolabs), and the PCR products were ligated using KLD Enzyme Mix (New England Biolabs). The ligated products were transformed into DH5α *Escherichia coli* cells. Mutagenesis was confirmed by Sanger sequencing analysis. The modified plasmid vectors were purified using a NucleoBond Xtra Midi EF kit (MN). RNA was synthesized using T7 RNA polymerase (New England Biolabs) in the presence of 1 μg of the purified plasmid and 4 mM NTPs (Jena Bioscience), purified using a Monarch RNA cleanup kit (New England Biolabs) and aliquoted into cryogenic vials before storage in liquid nitrogen.

### Human cell culture and transfection

HEK293T cells (LentX-293T, Takara) were maintained in Dulbecco’s modified Eagle’s medium (DMEM) supplemented with 10% heat-inactivated fetal bovine serum (FBS; Corning) and 1% penicillin–streptomycin at 37 °C in an incubator with a 5% CO_2_ atmosphere. Cell transfection was performed through either electroporation or lipofection. For electroporation, 2 μg of Cas12f1-, AsCas12a-, LbCas12a- or SpCas9-encoding plasmid vector was transfected together with 2 μg of gRNA-encoding DNAs into 70% confluent HEK293T cells in 24-well culture plates using a Neon transfection system (Invitrogen). The electroporation conditions were as follows: 1,300 V, 10 mA, 3 pulses. For lipofection, 15 μl of FuGene reagents (Promega) was mixed with 5 μg of Cas12f1-encoding plasmid vector + 5 μg of PCR amplicons in 70% confluent cells in 6-well culture plates and incubated for 15 min. The mixture (300 μl) was added to 1.5 ml DMEM in which 1 × 10^6^ cells had been plated 1 d before transfection, and cells were grown in the presence of the mixture for specified durations. All indel efficiency tests were performed with samples collected 5 d after transfection, except for experiments that require time-course trace of indel efficiency. After incubation, cells were harvested, and genomic DNA was prepared either manually using a PureHelix genomic DNA preparation kit (NanoHelix) or using a Maxwell RSC nucleic acid isolation workstation (Promega). Target information is compiled in Supplementary Table 1.

### Measurement of indel frequencies

Genomic DNA was isolated from HEK293T cells using a PureHelix genomic DNA preparation kit (NanoHelix). Target-specific primers were synthesized and used to amplify protospacer-containing regions with KAPA HiFi HotStart DNA polymerase (Roche) according to the manufacturer’s instructions. The resulting PCR amplicons were labeled with Illumina TruSeq HT dual indexes. The final PCR products were subjected to 150-bp paired-end sequencing using an Illumina iSeq 100. Indel frequencies were calculated by MAUND, which is available at https://github.com/ibs-cge/maund.

### Recombinant Cas12f1

The Cas12f1 gene was cloned into a modified pMAL-c2x vector (Addgene), in which the factor Xa cleavage sequence was changed into a tobacco etch virus (TEV) sequence. The vector construct was used to transform BL21(DE3) *E. coli* cells. An *E. coli* transformant colony was grown at 37 °C in lysogeny broth until the culture reached an optical density of 0.7. Cells were incubated at 30 °C overnight in the presence of 0.1 mM isopropylthio-β-d-galactoside and then collected by centrifugation at 3,500*g* for 30 min. Cells were resuspended in 20 mM Tris-HCl, pH 7.6, 500 mM NaCl, 5 mM 2-mercaptoethanol and 5% glycerol. Cell lysates were prepared by sonication followed by centrifugation at 15,000*g* for 30 min, and subsequent filtration through a 0.45-µm syringe filter (Millipore). The cleared lysates were loaded on to a Ni^2+^ affinity column (HisTrap HP 5 ml, GE Healthcare) using a fast protein liquid chromatography purification system (ÄKTA Purifier, GE Healthcare). The bound fractions were eluted with 20 mM Tris-HCl, pH 7.5 with 80–400 mM imidazole gradients. The eluted proteins were treated with 1 mg of TEV protease for 6 h. The cleaved proteins were purified on a heparin column with a linear gradient of 0.15–1.6 M NaCl. The recombinant Cas12f1 proteins were dialyzed against 20 mM Tris, pH 7.6, 150 mM NaCl, 5 mM 2-mercaptoethanol and 5% glycerol. The dialyzed proteins were again purified on a monoS column (GE Healthcare) with a linear gradient of 0.5–1.2 M NaCl. The selected fractions were pooled and dialyzed against 20 mM Tris, pH 7.6, 150 mM NaCl, 5 mM 2-mercaptoethanol and 5% glycerol. The concentration of the produced proteins was electropherometrically determined on a Coomassie Blue-stained sodium dodecylsulfate–polyacrylamide gel electrophoresis gel using bovine serum albumin as a standard.

### Analysis of potential off-target sites using Digenome-seq

Genome-wide off-target analysis was performed using the Digenome-seq method as described previously^44^. Briefly, genomic DNA was isolated from HEK293T cells and treated with ribonucleoprotein complexes formed by preincubating 10 μg of recombinant Cas12f1 or AsCas12a protein and engineered gRNA at 900 nM, respectively, at room temperature for 2 h. Digestion of genomic DNA was performed in a reaction buffer comprising 100 mM NaCl, 10 mM MgCl_2_, 100 μg ml^−1^ of bovine serum albumin, 50 mM Tris-HCl, pH 7.9 at 37 °C and 46 °C for AsCas12a and Cas12f, respectively, for 8 h. Digested genomic DNA was purified using a DNeasy Tissue kit (QIAGEN) after treatment with RNase A (50 μg ml^−1^). The purified genomic DNA was subjected to whole-genome sequencing (WGS) at a sequencing depth of 30× to 40× using a DNBSEQ-T7 Sequencer (MGI). A DNA cleavage score was assigned to each nucleotide position across the entire genome, using WGS data, according to an equation mentioned previously^44^. A cut-off value of 1.0 was assigned to identify potential off-target sites using the Digenome-seq program (https://github.com/chizksh/digenome-toolkit2) with an additional criterion of six or fewer mismatches with the on-target sequence. The identified potential off-target sites were validated in vivo by targeted deep-sequencing analysis after the treatment of HEK293T cells with Cas12f1 or AsCas12a and gRNAs.

### Droplet digital PCR

Genomic DNA was prepared from HEK293T cells treated with an AAV carrying CRISPR–SaCas9 or CRISPR–Cas12f1 vectors, which was linearized by Mlu I at 37 °C for 2 h. The linearized gDNA was mixed with QX200 ddPCR EvaGreen Supermix (BioRad, catalog no. 186–4033), 100 nM forward primers (5′-GGAAGGACTCATGACCACAGT-3′ for CEP290, 5′-GGAAGGACTCATGACCACAGT-3′ for glyceraldehyde 3-phosphate dehydrogenase (GAPDH)), 100 nM reverse primers (5′-TTCTTGTGGCAGTAAGGAGGA-3′ for CEP290, 5′-CAGTGAGCTTCCCGTTCAG-3′ for GAPDH); 20 μl of PCR reaction mixture and 70 μl of droplet-generation oil for EvaGreen (BioRad, catalog no. 1864005) were mixed into a well of DG8 Cartridges for QX100/QX200 Droplet Generator (BioRad, catalog no. 1864008). The cartridge was covered with DG8 Gaskets for QX100/QX200 Droplet Generator (BioRad, catalog no. 1863009). After the generation of droplets, 40 μl of droplets was pipetted into ddPCR 96-Well Plate (BioRad, catalog no. 12001925). PCR was performed in the heat-sealed plates under the conditions of initial denaturation 95 °C for 5 min, followed by 45 cycles of denaturation for 30 s at 95 °C, annealing/extension for 1 min at 64 °C, and signal stabilization for 5 min at 4 °C, 5 min at 90 °C, and ending at 4 °C with a ramping rate of 2 °C s^−1^. The signal measurements were performed in a QX200 Droplet Reader and data were analyzed using a QuantaSoft software.

### Quantitative analysis of gRNA expression

Either canonical or modified gRNA-encoding PCR amplicons (3 μg) were transfected into HEK293T cells using a Neon transfection system (Invitrogen), and cells were harvested after 2 d of transfection. Total RNA was prepared using a Maxwell RSC miRNA Tissue Kit (Promega, catalog no. AS1460). RNA was poly(adenylated) by incubating 5 μg of an RNA preparation with *E. coli* poly(A) polymerase (New England Biolabs, catalog no. M0276) at 37 °C for 30 min. RNA was purified with a Monarch RNA Cleanup Kit (New England Biolabs, catalog no. T2050). Then, 500 μg of poly(A)-tailed RNA was reverse transcribed using SuperScript IV Reverse Transcriptase (Invitrogen) in the presence of an RT-specific primer that carries the T6 sequence at its 3′ terminus and an adapter sequence. The gRNA was PCR amplified using adapter- and gRNA-specific primers. PCR products were resolved on 2% agarose gels.

### In vitro DNA cleavage

Plasmid vectors harboring a protospacer sequence were constructed for DNA-cleavage assay. Plasmid vectors, 2 μg, were incubated with 10 μM recombinant Cas12f1 in the presence of 6 μM gRNAs at 37 or 46 °C for 2 h. ApaI (New England Biolabs), 1 μl, was treated in 50 μl of reaction samples at 37 °C for 40 min as a control. The incubated samples were treated with RNase at 10 units ml^−1^ for 1 h. The incubated samples were resolved on 2% agarose gels.

### Production of AAV vectors and transduction

The human codon-optimized Cas12f1 and sgRNA sequences were cloned into AAV vector plasmids with inverted terminal repeats. The Cas12f1 gene was accompanied by a nuclear localization signal and linked to the eGFP gene via a self-cleaving T2A sequence. Cas12f1 and sgRNA transcription were driven by the chicken β-actin and the U6 promoters, respectively. To produce rAAV2 vectors, HEK293T cells were transfected with pAAV-ITR-sgRNA-Cas12f1 or pAAV-ITR-sgRNA-SaCas9, pAAVED2/2 and helper plasmid. HEK293T cells were cultured in DMEM with 2% FBS. Recombinant pseudotyped AAV vector stocks were generated using polyethylenimine coprecipitated with PEIpro (Polyplus transfection) and triple transfection with plasmids at an equal molar ratio in HEK293T cells. After 72 h of incubation, cells were lysed and particles were purified by iodixanol (Sigma-Aldrich) step-gradient ultracentrifugation. The number of vector genomes was determined by quantitative (q)PCR. HEK293T cells were infected with rAAV2-Cas12f1-sgRNA or rAAV2-SaCas9-sgRNA at different multiplicities of infection as determined by qPCR. The transduced cells were maintained in DMEM with 2% FBS for up to 2 weeks. At different time points, cells were collected for isolation of genomic DNA.

### Statistical analysis

Statistical significance tests were performed using SigmaPlot software (v.14.0) through a two-tailed Student’s *t*-test, Mann–Whitney *U*-test, Welch’s *t*-test or one-way analysis of variance (ANOVA) test. *P* values <0.05 were considered significant. Data points in box and dot plots represent the full range of values, and boxes span the interquartile range (25th to 75th percentiles). Median and average values are indicated by horizontal lines. The error bars in all dot and bar plots show the s.d. and were plotted using SigmaPlot (v. 14.0). We did not predetermine sample sizes based on statistical methods. Large-scale validations were performed blinded to information with respect to the Cas type. For all experimental results, the *n* is reported in the accompanying figure legend.

### Reporting Summary

Further information on research design is available in the Nature Research Reporting Summary linked to this article.

## Online content

Any methods, additional references, Nature Research reporting summaries, source data, supplementary information, acknowledgements, peer review information; details of author contributions and competing interests; and statements of data and code availability are available at 10.1038/s41587-021-01009-z.

## Supplementary information

Supplementary Information Supplementary Figs. 1–10.

Reporting Summary

Supplementary Table 1 Target information used in indel efficiency tests.

Supplementary Table 2 Target information on a large-scale validation for SpCas9, AsCas12a and Cas12f.

Supplementary Table 3 Lists of off-target sites for Cas12a and Cas12f.

Supplementary Table 4 Potential off-target sites identified by Digenome-seq analysis.

Supplementary Table 5 SgRNA-encoding sequences for engineered Cas12f systems.

Supplementary Data Source data for Supplementary Figs. 1, 2, 3, 4 and 7.

## Data Availability

Data that support the findings of the present study are available within the article and its Supplementary Information. The WGS data were deposited at the National Center for Biotechnology Information Sequence Read Archive under accession no. PRJNA699382. All other data that support the findings of the present study and plasmid vectors are available from the corresponding author upon request. Unprocessed gel images are provided in Supplementary Fig. 10. Source data are provided with this paper.

## Source data

Source Data Fig. 1 Statistical source data for Fig. 1.
Source Data Fig. 2 Statistical source data for Fig. 2.
Source Data Fig. 3 Statistical source data for Fig. 3.
Source Data Fig. 4 Statistical source data for Fig. 4.

## Code availability

The custom codes used for Digenome-seq analysis can be found at GitHub (https://github.com/chizksh/digenome-toolkit2).
