# Supplementary material for: Efficient CRISPR editing with a hypercompact Cas12f1 and engineered guide RNAs delivered by adeno-associated virus
Source: Nat Biotechnol. 2021 Sep 2;40(1):94–102. doi: 10.1038/s41587-021-01009-z (PMC8763643; doi:10.1038/s41587-021-01009-z)

nature portfolio

https://doi.org/10.1038/s41587-021-01009-z

Supplementary information

Efficient CRISPR editing with a hypercompact Cas12f1 and engineered guide RNAs delivered by adeno-associated virus

In the format provided by the authors and unedited

Supplementary Information

Efficient CRISPR editing with a hypercompact Cas12f1 and engineered guide RNAs delivered by adeno-associated virus

Do Yon Kim, Jeong Mi Lee, Su Bin Moon, Hyun Jung Chin, Seyeon Park, Youjung Lim, Daesik Kim, Taeyoung Koo, Jeong-Heon Ko, Yong-Sam Kim

Supplementary Figure 1. Optimized tracrRNA and crRNA sequences at the penta-uridinylate site (MS1).

Supplementary Figure 2. Addition of a polyuridinylated 3’-overhang (MS2) to the MS1 crRNA further enhances the indel-inducing efficiency of Cas12f1.

Supplementary Figure 3. Truncated crRNA and tracrRNA (MS3, MS4, and MS5) synergistically potentiate MS1/MS2 sgRNA.

Supplementary Figure 4. Possible causes of the increased Cas12f-mediated indel frequencies induced by the gRNA modifications.

Supplementary Figure 5. Fully optimized versions of gRNAs for Cas12f_ge3.0, _4.0, and 4.1.

Supplementary Figure 6. Analysis of indel patterns generated by Cas12f.

Supplementary Figure 7. Feasibility tests for Cas12f-mediated genome editing through AAV2 delivery.

Supplementary Figure 8. Long-deletion profiles at an on-target site upon indel formation.

Supplementary Figure 9. Nucleotide sequence for the human codon-optimized Cas12f1 construct.

Supplementary Figure 10. Unprocessed gel images for Figure 3d and Extended data Figure 4b and 4d

Supplementary Table 1. Target information used in indel efficiency tests.

Supplementary Table 2. Target information on a large-scale validation for SpCas9, AsCas12a, and Cas12f.

Supplementary Table 3. Lists of off-target sites for Cas12a and Cas12f.

Supplementary Table 4. Potential off-target sites identified by Digenome-seq analysis.

Supplementary Table 5. Single-guide RNA-encoding sequences for engineered Cas12f systems.

Supplementary Figure 1. Optimized tracrRNA and crRNA sequences at the penta-uridinylate site (MS1)

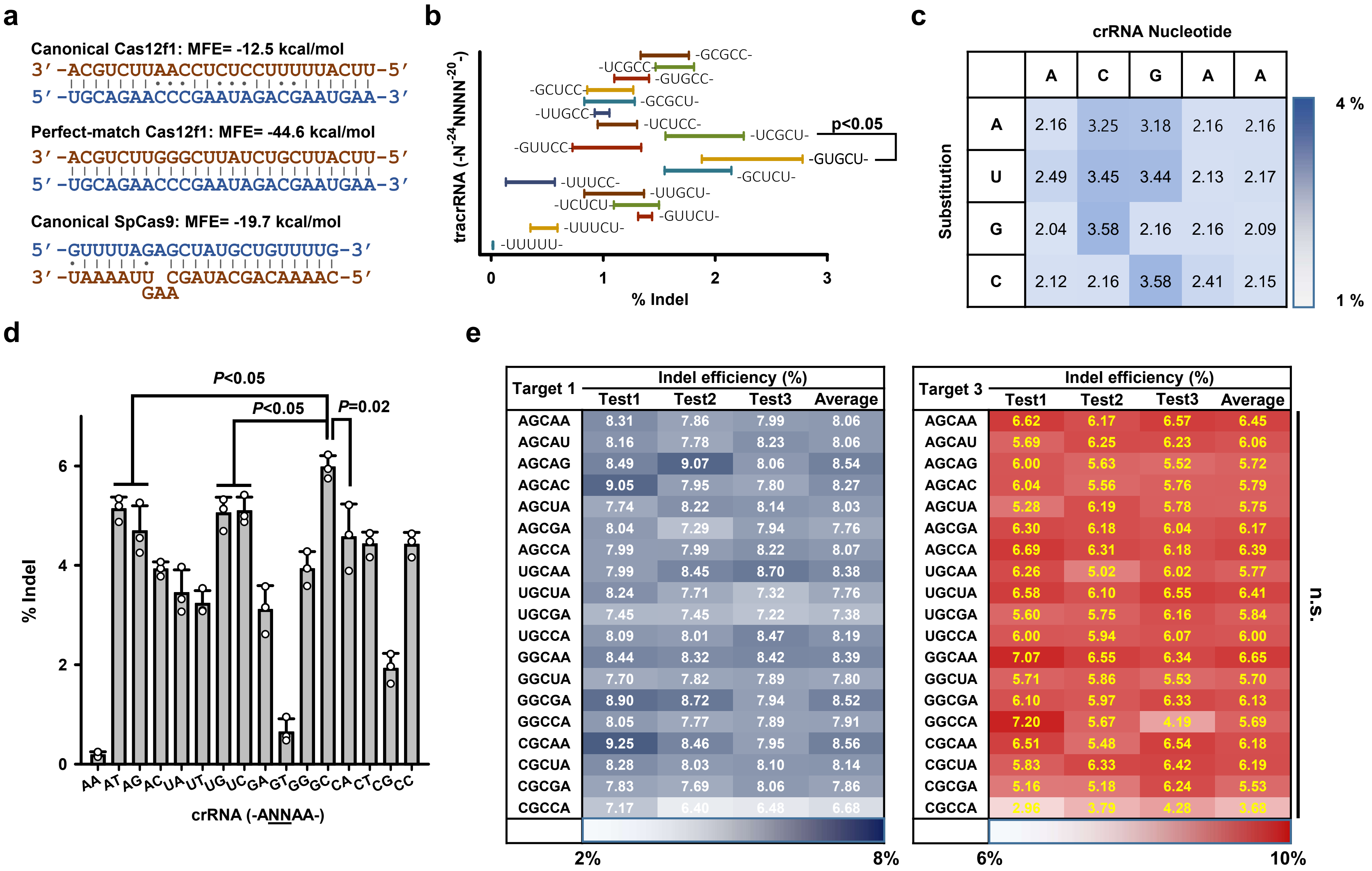

Supplementary Figure 1. Optimized tracrRNA and crRNA sequences at the penta-uridinylate site (MS1). a. The base-paring strength in the tracrRNA-crRNA complementary region was predicted by calculating the minimal free energy (MFE) *in silico*. The tracrRNA and crRNA for Cas12f1 carry energetically unfavorable sequences for hybridization because a stretched base pair is not formed due to the frequent occurrence of mismatches between the two regions. The predicted MFE for canonical tracrRNA-crRNA base pairing was ~12.5 kcal/mol, which is quite a low binding energy, compared to that for a perfectly matched sequence (-44.6 kcal/mol). The tracrRNA-crRNA binding energy for canonical Cas12f1 was even lower than that for SpCas9. We assumed that the canonical gRNA structure *per se* might dampen the effects of our gRNA engineering and thus would not reflect improvements of Cas12f1 efficiency caused by gRNA engineering. Therefore, we began gRNA engineering using the sgRNA form. **b**. The optimal sequence at the MS1 site in the tracrRNA was investigated by fixing a cytidine (C) in place of the uridine (U) at the -21 position in the tracrRNA. Sixteen combinatorial sequences at the penta-uridinylate site were compared with respect to indel frequencies induced in HEK293T cells. The plot indicates the 25%-75% percentile range in a box-and-whisker plot. n=3. 'GUGCU' was determined to be the optimal MS1 engineering in the tracrRNA. **c**. Changes in indel frequencies were monitored after varying the crRNA sequence at the MS1 site. Substitutions were made at each position in the original 'ACGAA' sequence, and significant improvements were observed for the 'C' and 'G' sites. **d**. The '–CG-' sequence in the crRNA was varied to identify the optimal sequence for the counterpart to the tracrRNA sequence in the crRNA. A substitution of –CG- with –GC- enabled an increase in indel frequency by *ca.* 3-fold. n=3. **e**. The final optimized sequence at the MS1 site in the crRNA was determined to be 'AGCAA'. In line with the results in Supplementary Fig. 1c, a substitution in the last position to obtain 'ACGAA' in the crRNA did not affect Cas12f1-mediated indel frequencies, and thus 'A' was fixed at this position as in the original sequence. The remaining two positions were compared with 16 combinatorial sequences, but we did not observe any dramatic changes in indel frequencies, except in the case of 'CGCGA'. That is, changing 'CG' to 'GC' was sufficient to derive the optimal sequence in the crRNA at the MS1 site. In conclusion, the penta-uridinylate site (MS1) was engineered to 5'-GUGCU-3' in the tracrRNA and 5'-AGCAA' in the crRNA and the MS1 engineering alone led to an increase in indel frequency by ~600 to 800-fold. n=3. *P*-values were derived by two-sided, student's t-test. n.s., not significant. Error bars represent SD.

Supplementary Figure 2. Addition of a polyuridinylated 3'-overhang (MS2) to the MS1 crRNA further enhances the indel-inducing efficiency of Cas12f1

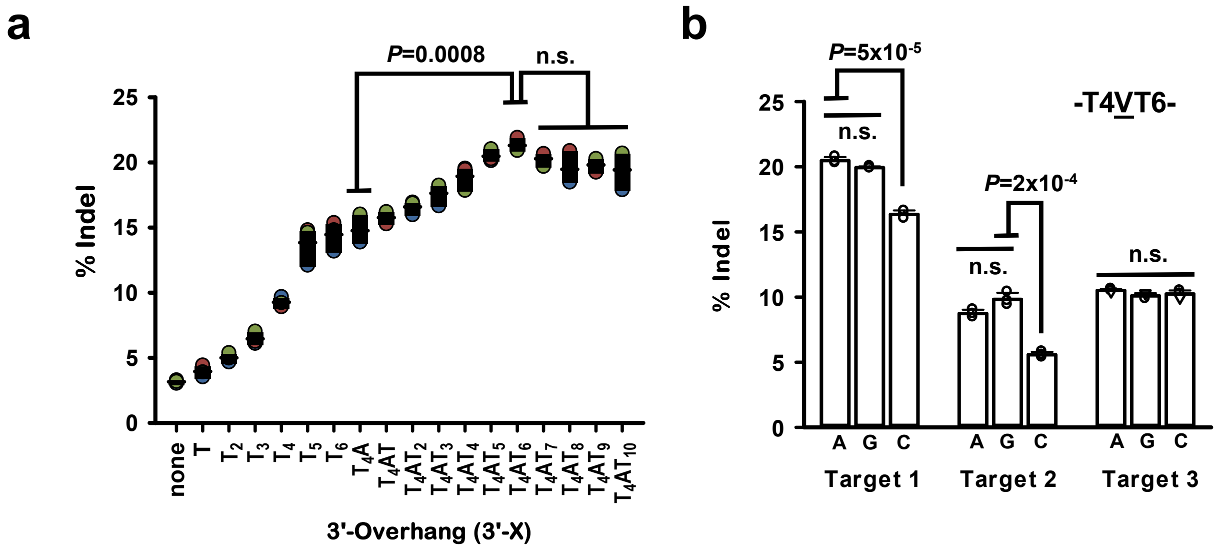

Supplementary Figure 2. Addition of a polyuridinylated 3'-overhang (MS2) to the MS1 crRNA further enhances the indel-inducing efficiency of Cas12f1. a. Increased Cas12f1-mediated indel frequencies were obtained by the attachment of a polyuridinylated sequence to the penta U-modified crRNA (MS1). Increasing the number of thymidines in the gRNA-encoding template up to four led to sequential increases in Cas12f1-mediated indel frequencies, but further additions were not effective due to the creation of a termination sequence for the U6 promoter. To obviate the termination cue, an adenylate was inserted after the T4 sequence and more thymidines were added. Finally, the addition of T4AT6 resulted in the highest Cas12f1-mediated indel frequencies. **b**. To derive an optimal engineered sequence at the MS2 site, the adenylate in T4A̲T6 was substituted with cytidine or guanidine. Experiments employing three endogenous targets indicated that the T4AT6 and T4GT6 sequences resulted in similar indel frequencies, whereas T4CT6 was not optimal for some of the tested targets. n=3. All error bars represent SD. *P*-values were derived by a two-sided student's t-test; n.s.; not significant.

Supplementary Figure 3. Truncated crRNA and tracrRNA (MS3, MS4, and MS5) synergistically potentiate MS1/MS2 sgRNA

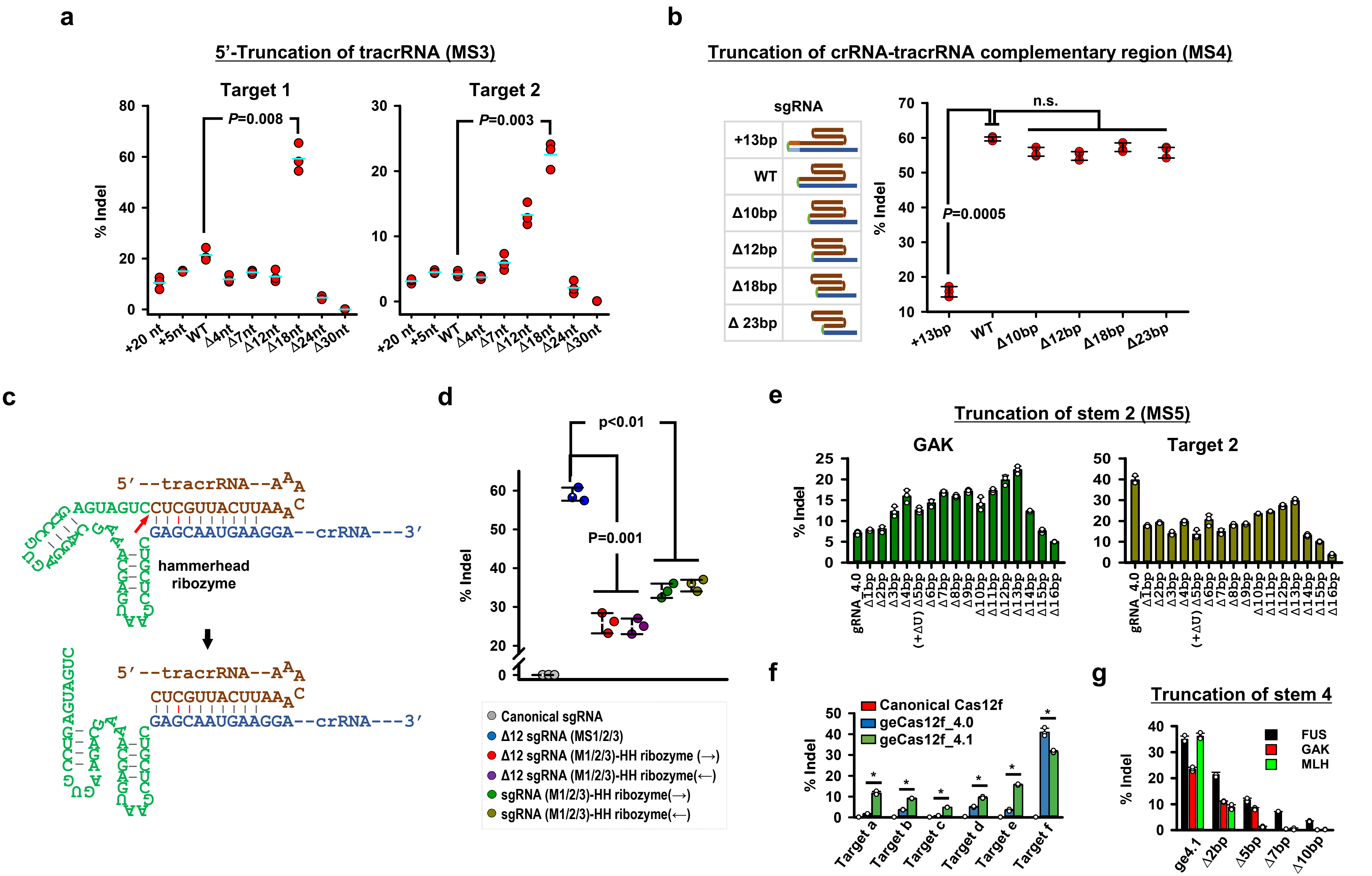

Supplementary Figure 3. Truncated crRNA and tracrRNA (MS3, MS4, and MS5) synergistically potentiate MS1/MS2 sgRNA. a. Truncation of the 5'-terminal region of the tracrRNA increases Cas12f1-mediated indel frequencies. Whereas increasing the length of the 5'-terminus of the tracrRNA diminished the effects of MS1 and MS2 modifications, 5'-truncations of the tracrRNA further increased indel frequencies. In particular, truncation of 18-nt led to a dramatic surge in indel frequencies at two independent targets. n=3. **b**. Truncation of the crRNA-tracrRNA complementary region also affected indel frequencies. The elongation of the complementary region resulted in a dramatic decrease in the frequency. In contrast, truncation of the complementary sequence did not yield significant decreases in indel frequencies. In particular, an 18-nt truncation led to almost comparable indel frequencies as the uncleaved sgRNA. n=3. **c**. Elongation of the crRNA-tracrRNA complementary region was attempted by substituting the 'GAAA' tetraloop with a hammerhead (HH) ribozyme. The incorporation of the HH sequence result in an asymmetric lengths in the crRNA and tracrRNA through self-cleavage. Changing the direction of the HH sequence can be used to differentially regulate the lengths of the 3'- and 5'-terminal regions of the tracrRNA and crRNA, respectively. **d**. An increase in either the tracrRNA or crRNA through incorporation of a HH ribozyme reduced engineered Cas12f1-mediated indel frequencies. The arrow indicates the direction of the incorporated hammerhead ribozyme sequence. n=3. **e**. gRNA engineering in the stem 2 region was effective in further increasing indel frequencies at GAK site but not at Target 2, compared to gRNA_4.0. Delta-n refers to a paired deletion of two nucleotides from both sides of the stem loop. n=3. **f**. Cas12f_ge4.1 induced higher indel frequencies for some of the tested targets, compared to Cas12f_ge4.0. Cas12f_ge4.1 was effective in a target-dependent manner, particularly for targets with low indel frequencies. **P*-values are a, 5×10^{-5}; b, 3×10^{-6}; c, 3×10^{-7}; d, 2×10^{-4}; e, 3×10^{-6}; f, 0.002. **g**. Truncation of stem 4 region of tracrRNA. n=3. *P*-values were derived by a two-sided student's t-test; n.s.; not significant. All error bars represent SD.

Supplementary Figure 4. Possible causes of the increased Cas12f-mediated indel frequencies induced by the gRNA modifications

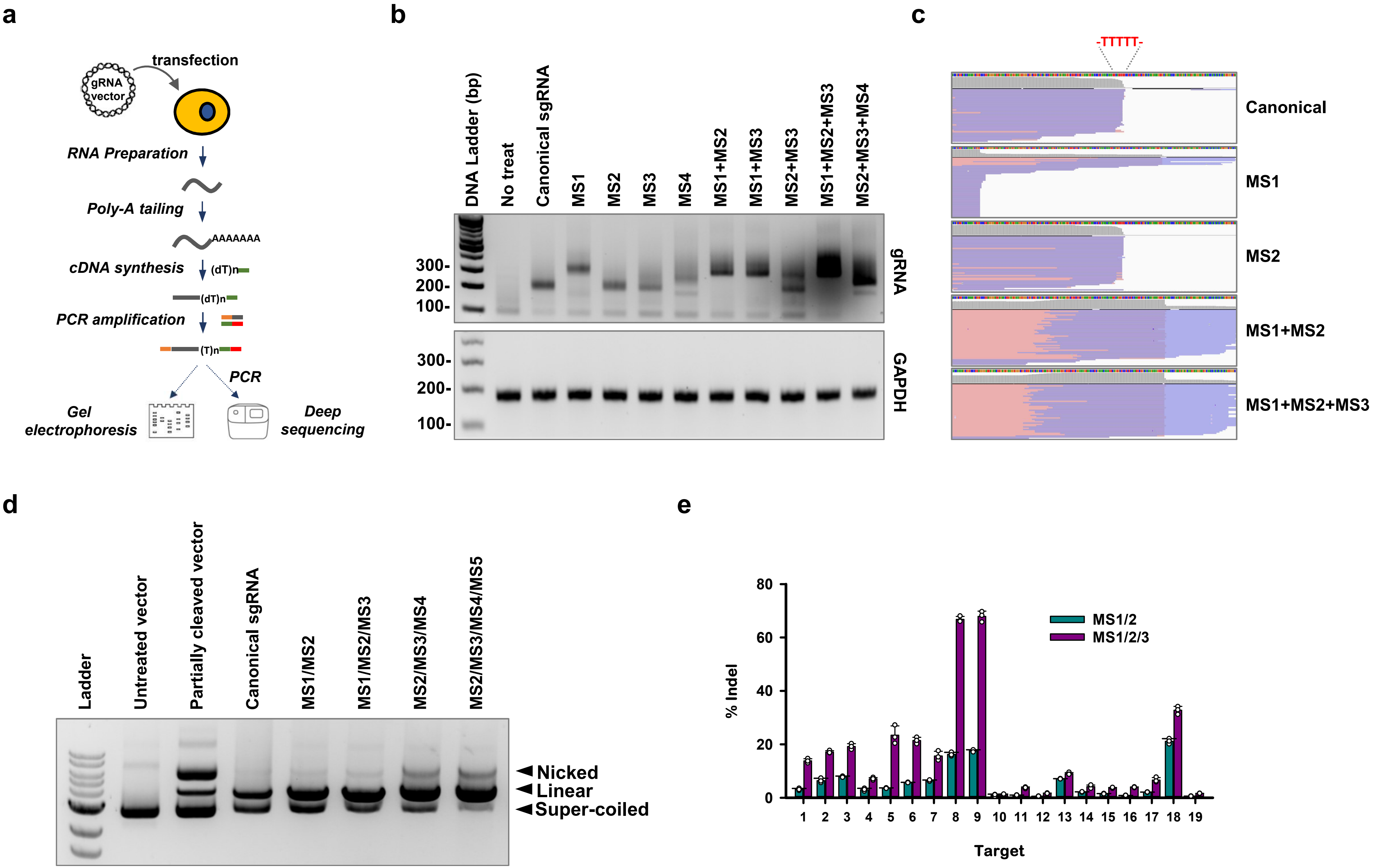

Supplementary Figure 4. Possible causes of the increased Cas12f-mediated indel frequencies induced by the gRNA modifications. a. Scheme for the analysis of quantitative and qualitative changes in gRNA caused by each step of gRNA engineering. Plasmid vectors encoding canonical or engineered gRNAs were transfected into HEK293T cells, from which total RNA was prepared. The RNA samples were subjected to poly-A tailing using poly(A) polymerase, and cDNA was synthesized using the poly-A tailed RNA as a template using reverse transcriptase and oligo(dT) with an adapter sequence. Finally, RNA was PCR-amplified and the amplified PCR products were analyzed by agarose gel electrophoresis and deep sequencing. **b**. Expression patterns of canonical and engineered gRNAs assayed using agarose gel electrophoresis. The canonical sgRNA was observed to be smaller in size than expected, and MS1 engineering was responsible for recovery of the full-sized gRNA. Besides causing an increase in gRNA-Cas affinity, the MS2 engineering appears to stabilize the expression of full-length sgRNA. It is noteworthy that MS3 modifications also increased expression of gRNA. The *GAPDH* gene was used as a loading control (representative data for three experiments). **c**. IGV images mirroring the expression patterns of canonical and engineered gRNAs. Expression of the canonical gRNA was terminated in the penta-uridinylate region (MS1 site), and MS1 engineering partly enabled the production of full-length gRNA. Although the MS2 modification itself did not rescue the impaired expression, it stabilized the expression of full-length gRNAs when combined with the MS1 engineering. MS3 engineering did not cause significant changes in the expression pattern. **d**. Increased *in vitro* cleavage of plasmid DNA carrying a target sequence induced by MS1-MS5 engineering at 37°C. The linearized form of the plasmid was dramatically increased by MS1/MS2 engineering. MS3, MS4 and MS5 modifications also increased *in vitro* DNA cleavage activity (representative data for five experiments). **e**. Validation of the effect of MS3 engineering on the increased indel frequencies *in vivo*. Almost all targets tested showed a drastic increase in indel frequencies, with an average fold increase of 3.12. n=3. Error bars represent SD.

Supplementary Figure 5. Fully optimized versions of gRNAs for Cas12f_ge3.0, _ge4.0, and ge4.1

	Structure	Size (nt)	Efficiency range* (Average ± SD)	gRNA engineering
**Cas12f_ge3.0**	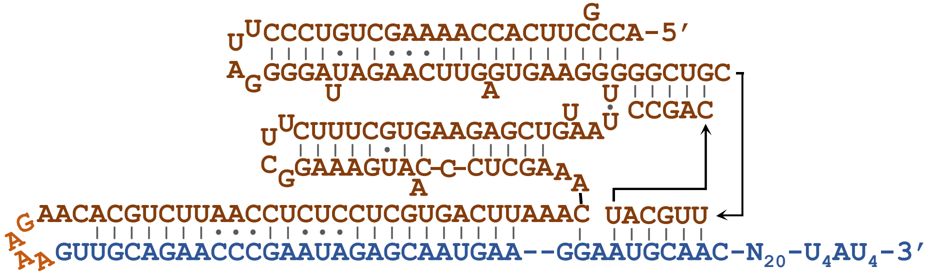	tracrRNA: 141<br>Loop: 4<br>crRNA repeat: 37<br>Spacer: 20<br>U-tail: 9<br>**Total: 211**	0 - 74.2%<br>(13.68 ± 20.70)	MS1<br>MS2<br>MS3<br>(single guide RNA)
**Cas12f_ge4.0**	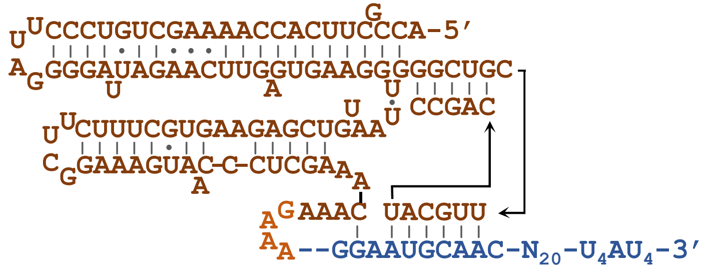	tracrRNA: 113<br>Loop: 4<br>crRNA repeat: 10<br>Spacer: 20<br>U-tail: 9<br>**Total: 156**	0 - 75.3%<br>(20.76 ± 16.19)	MS2<br>MS3<br>MS4<br>(single guide RNA)
**Cas12f_ge4.1**	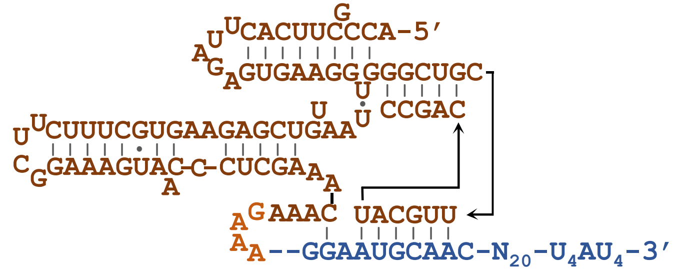	tracrRNA: 86<br>Loop: 4<br>crRNA repeat: 10<br>Spacer: 20<br>U-tail: 9<br>**Total: 129**	0 – 85.8%<br>(26.00 ± 19.81)	MS2<br>MS3<br>MS4<br>MS5<br>(single guide RNA)

*** Efficiency range was derived from the large-scale analysis (Fig. 2b).**

Brief Description of gRNA modifications:

MS1: Correction of an internal penta-uridinylate region into GUGCU in tracrRNA and AGCAA in crRNA

MS2: Addition of a 3’- poly-uridinylate sequence (U4AU4) in crRNA

MS3: Truncation of 20-nt at the 5’-terminus in tracrRNA

MS4: Truncation of tracrRNA-crRNA complementary sequence

MS5: Partial truncation of stem 2 region in tracrRNA

Supplementary Figure 5. Fully optimized versions of gRNAs for Cas12f_ge3.0, _4.0, and 4.1. The gRNA for Cas12f_ge3.0 was derived from the combined MS1, MS2, and MS3 engineering steps. The tracrRNA and crRNA were connected with a 'GAAA' tetraloop, creating an efficient sgRNA. To generate the gRNA for Cas12f_ge4.0, the MS2, MS3, and MS4 engineering steps were in performed in a sgRNA. Cas12f_ge4.1 was further developed from Cas12f_ge4.0 by removing the part of stem 2 in the tracrRNA spanning from A(-130) to U(-102). Cas12f_ge4.1 was effective in further increasing the indel frequency in a significant portion of targets, compared to Cas12f_ge4.0. gRNA-4.1 has more favorable properties as a gene therapeutic agent, in that it is significantly smaller (131 nt) and results in a higher indel frequency in most of the tested targets (78%), compared to Cas12f_3.0 and _4.0.

Supplementary Figure 6. Analysis of indel patterns generated by Cas12f

a

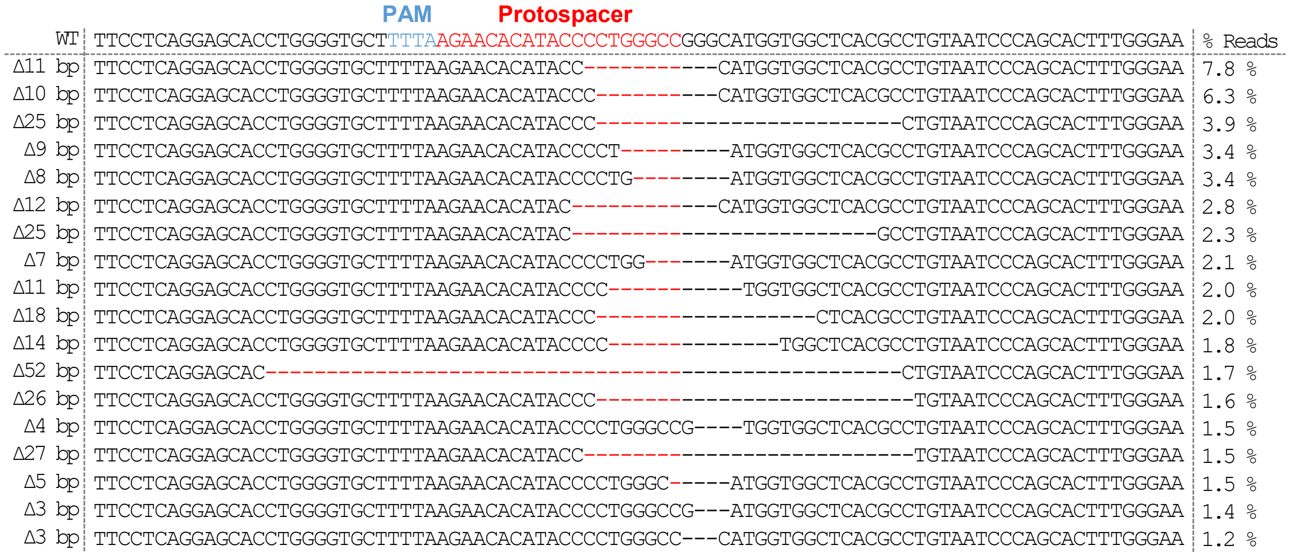

b

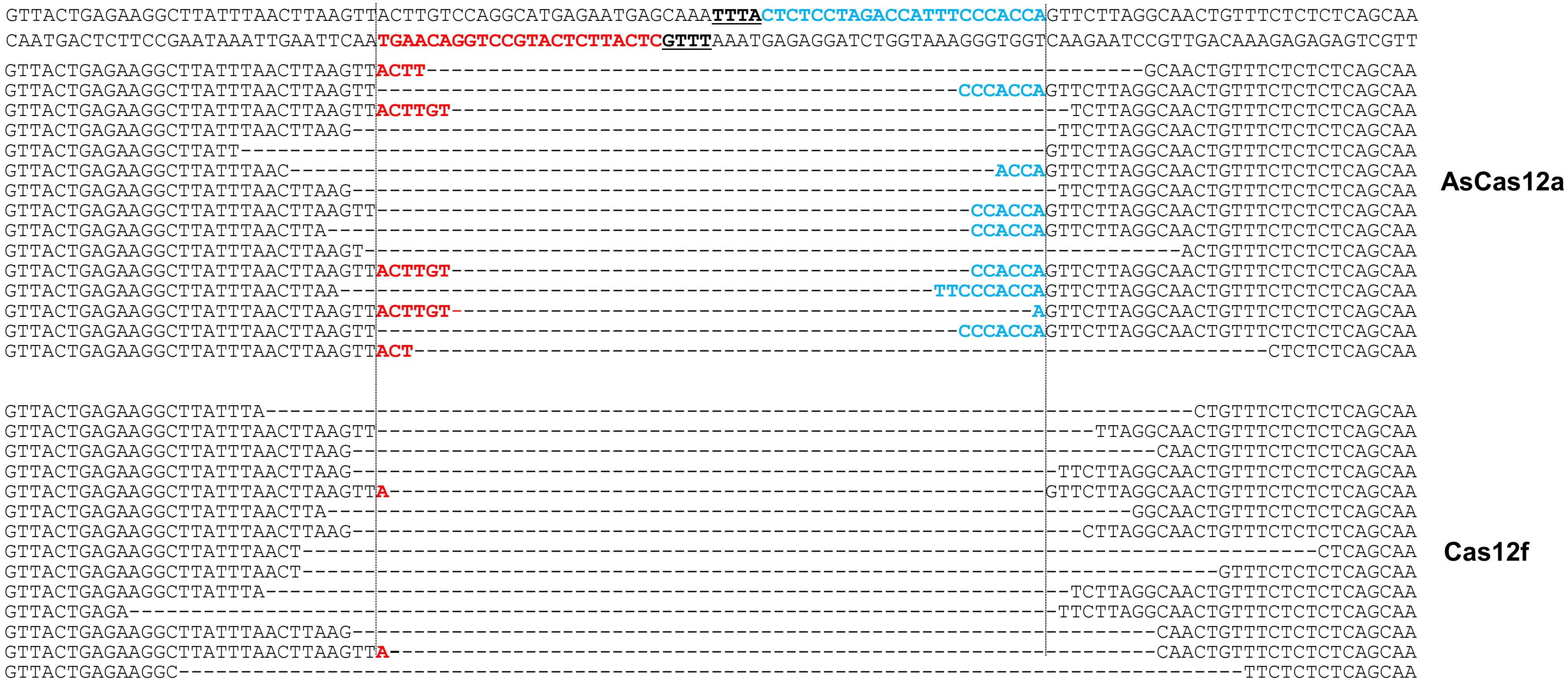

Supplementary Figure 6. Analysis of indel patterns generated by Cas12f. a. Indel patterns in a target site in HEK293T cells transfected with Cas12f_ge4.1. The indel patterns were obtained by deep sequencing analysis of PCR amplicons containing a target site, revealing that almost all of the reads show deletions that affect the protospacer region. In particular, such a property is obvious in reads with a relatively high percentage of indels; deletions that did not alter the protospacer sequence occurred in only a small fraction of these reads. This pattern provides indirect evidence that multiple Cas12f-mediated cleavage events occur, during which a sequence containing an initial short indel is further cleaved, culminating in the generation of a long-deletion mutation over time. **b**. Differences between the cleavage patterns induced by AsCas12a and Cas12f. We selected a locus containing a target for each enzyme; the targets were located close to each other and were oriented in opposite directions, and each carried a TTTA and a TTTG PAM sequence. The targets were subjected to AsCas12a- and Cas12f-mediated cleavage to create indel mutations. In contrast to the indel pattern induced by AsCas12a, which preserves a protospacer sequence in a fraction of reads, after Cas12f exposure the protospacer sequences were absent from all sequence reads. These results strongly indicate that Cas12f induces relatively long deletion mutations through multiple dsDNA cleavages outside the protospacer. It is interesting to note that insertions were rarely observed for Cas12f-mediated genome editing.

Supplementary Figure 7. Feasibility tests for Cas12f-mediated genome editing through AAV2 delivery

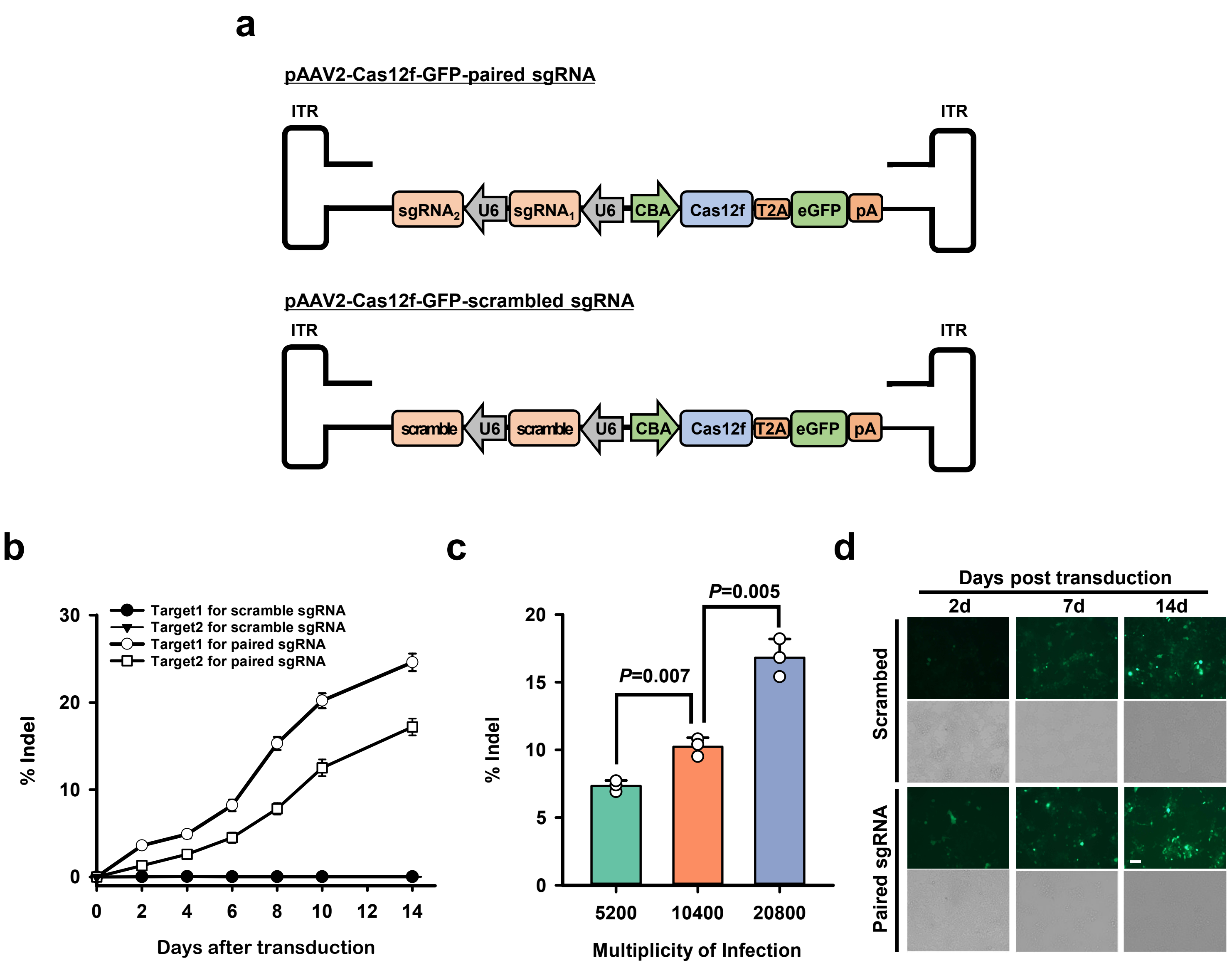

Supplementary Figure 7. Feasibility tests for Cas12f-mediated genome editing through AAV2 delivery. a. The DNA structure of rAAV2 vectors carrying the Cas12f_ge4.1 system. Expression of the two sgRNAs is controlled by individual U6 promoters. The sgRNAs targeted Targets 1 and 2 used throughout this study. gRNAs with scrambled sequences were used as a control. **b**. Time-course of indel generation following transduction of HEK293T cells with rAAV2 carrying the Cas12f system. For transduction, $2X10^6$ HEK293T cells were plated and treated with $1.5X10^7$ vg/ml rAAV2 particles. Cells were periodically subcultured and viral particles were added at the same MOI 24 h after subculture. n=3. **c**. Dependence of the Cas12f-mediated indel frequency on the concentration of viral particles. Cells were transduced with given concentrations of AAV2 particles and grown for 5 days without further addition of viral particles. n=3. **d**. Fluorescence images of HEK293T cells transduced with Cas12f-carrying viral particles (representative images for three experiments; scale bar, 100μm). *P*-values were derived by a two-sided student's t-test. All error bars represent SD.

Supplementary Figure 8. Long-deletion profiles at an on-target site upon indel formation

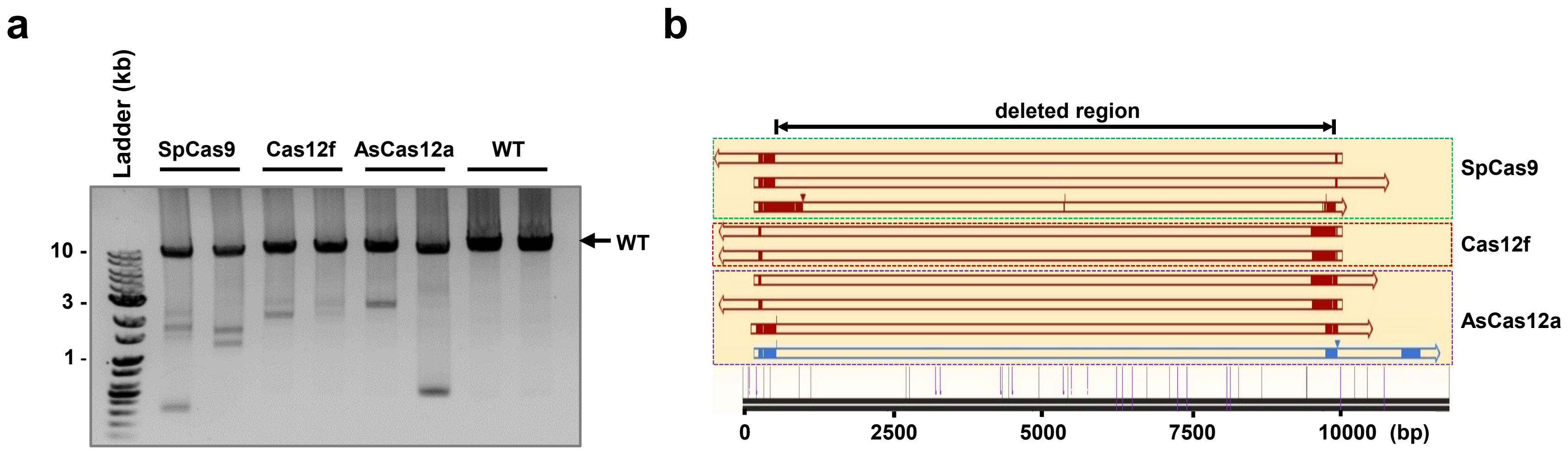

Supplementary Figure 8. Long-deletion profiles at an on-target site upon indel formation. a. An on-target site (5'-TTTACTCTCCTAGACCATTTCCCACCAGTT-3') was subjected to indel formation with either SpCas9, Cas12f_ge4.1, or AsCas12a. After 5 days post transfection, genomic DNA was prepared from HEK293T cells. A 10-kb genomic region spanning the on-target site was amplified by 3 rounds of PCR reactions, each of which used different sets of primers for specific amplification. An agarose gel image indicates the presence of long-deletion fragments for all the Cas effector proteins (representative data for three experiments). **b**. The sanger sequencing analyses confirmed the presence of long-deletion (longer than 9 kb) products for SpCas9, Cas12f, and AsCas12a.

Supplementary Figure 9. Nucleotide sequence for the human codon-optimized Cas12f1 construct

CCAAAGAAGAAGCGGAAGGTCGGTATCCACGGAGTCCCAGCAGCCATGGCCAAGAACACAATTACAAAGACACTGAAGCTGAGGATCGTGAGACCATACAACAGCGCTGAGGTCGAGAAG
ATTGTGGCTGATGAAAAGAACAACAGGGAAAAGATCGCCCTCGAGAAGAACAAGGATAAGGTGAAGGAGGCCTGCTCTAAGCACCTGAAAGTGGCCGCCTACTGCACCACACAGGTGGAG
AGGAACGCCTGTCTGTTTTGTAAAGCTCGGAAGCTGGATGATAAGTTTTACCAGAAGCTGCGGGGCCAGTTCCCCGATGCCGTCTTTTGGCAGGAGATTAGCGAGATCTTCAGACAGCTG
CAGAAGCAGGCCGCCGAGATCTACAACCAGAGCCTGATCGAGCTCTACTACGAGATCTTCATCAAGGGCAAGGGCATTGCCAACGCCTCCTCCGTGGAGCACTACCTGAGCGACGTGTGC
TACACAAGAGCCGCCGAGCTCTTTAAGAACGCCGCTATCGCTTCCGGGCTGAGGAGCAAGATTAAGAGTAACTTCCGGCTCAAGGAGCTGAAGAACATGAAGAGCGGCCTGCCCACTACA
AAGAGCGACAACTTCCCAATTCCACTGGTGAAGCAGAAGGGGGGCCAGTACACAGGGTTCGAGATTTCCAACCACAACAGCGACTTTATTATTAAGATCCCCTTTGGCAGGTGGCAGGTC
AAGAAGGAGATTGACAAGTACAGGCCCTGGGAGAAGTTTGATTTCGAGCAGGTGCAGAAGAGCCCCAAGCCTATTTCCCTGCTGCTGTCCACACAGCGGCGGAAGAGGAACAAGGGGTGG
TCTAAGGATGAGGGGACCGAGGCCGAGATTAAGAAAGTGATGAACGGCGACTACCAGACAAGCTACATCGAGGTCAAGCGGGGCAGTAAGATTGGCGAGAAGAGCGCCTGGATGCTGAAC
CTGAGCATTGACGTGCCAAAGATTGATAAGGGCGTGGATCCCAGCATCATCGGAGGGATCGATGTGGGGGTCAAGAGCCCCCTCGTGTGCGCCATCAACAACGCCTTCAGCAGGTACAGC
ATCTCCGATAACGACCTGTTCCACTTTAACAAGAAGATGTTCGCCCGGCGGAGGATTTTGCTCAAGAAGAACCGGCACAAGCGGGCCGGACACGGGGCCAAGAACAAGCTCAAGCCCATC
ACTATCCTGACCGAGAAGAGCGAGAGGTTCAGGAAGAAGCTCATCGAGAGATGGGCCTGCGAGATCGCCGATTTCTTTATTAAGAACAAGGTCGGAACAGTGCAGATGGAGAACCTCGAG
AGCATGAAGAGGAAGGAGGATTCCTACTTCAACATTCGGCTGAGGGGGTTCTGGCCCTACGCTGAGATGCAGAACAAGATTGAGTTTAAGCTGAAGCAGTACGGGATTGAGATCCGGAAG
GTGGCCCCCAACAACACCAGCAAGACCTGCAGCAAGTGCGGGCACCTCAACAACTACTTCAACTTCGAGTACCGGAAGAAGAACAAGTTCCCACACTTCAAGTGCGAGAAGTGCAACTTT
AAGGAGAACGCCGATTACAACGCCGCCCTGAACATCAGCAACCCTAAGCTGAAGAGCACTAAGGAGGAGCCCAAAAGGCCGGCGGCCACGAAAAAGGCCGGCCAGGCAAAAAAGAAAAAG
GAATTCGGCAGTGGAGAGGGCAGAGGAAGTCTGCTAACATGCGGTGACGTCGAGGAGAAT**CCTGGCCCA**GTGAGCAAGGGCGAGGAGCTGTTCACCGGGGTGGTGCCCATCCTGGTCGAG
CTGGACGGCGACGTAAACGGCCACAAGTTCAGCGTGTCCGGCGAGGGCGAGGGCGATGCCACCTACGGCAAGCTGACCCTGAAGTTCATCTGCACCACCGGCAAGCTGCCCGTGCCCTGG
CCCACCCTCGTGACCACCCTGACCTACGGCGTGCAGTGCTTCAGCCGCTACCCCGACCACATGAAGCAGCACGACTTCTTCAAGTCCGCCATGCCCGAAGGCTACGTCCAGGAGCGCACC
ATCTTCTTCAAGGACGACGGCAACTACAAGACCCGCGCCGAGGTGAAGTTCGAGGGCGACACCCTGGTGAACCGCATCGAGCTGAAGGGCATCGACTTCAAGGAGGACGGCAACATCCTG
GGGCACAAGCTGGAGTACAACTACAACAGCCACAACGTCTATATCATGGCCGACAAGCAGAAGAACGGCATCAAGGTGAACTTCAAGATCCGCCACAACATCGAGGACGGCAGCGTGCAG
CTCGCCGACCACTACCAGCAGAACACCCCCATCGGCGACGGCCCCGTGCTGCTGCCCGACAACCACTACCTGAGCACCCAGTCCGCCCTGAGCAAAGACCCCAACGAGAAGCGCGATCAC
ATGGTCCTGCTGGAGTTCGTGACCGCCGCCGGGATCACTCTCGGCATGGACGAGCTGTACAAGGAATTCTAA

Red letter: Nuclear localization signal
Blue letter: Human codon-optimized Cas14a1 CDS
Green letter: T2A
Black letter: linker
Purple letter: eGFP CDS

Supplementary Figure 9. Nucleotide sequence for the human codon-optimized Cas12f1 construct. The whole sequence consists of a nuclear localization signal, human codon-optimized Cas14a1 CDS, eGFP CDS, T2A, and a linker sequences, which were noted in different colors.

Supplementary Figure 10. Unprocessed gel images for Figure 3d and Extended data Figure 4b and 4d

a. Unprocessed gel image for Figure 3d

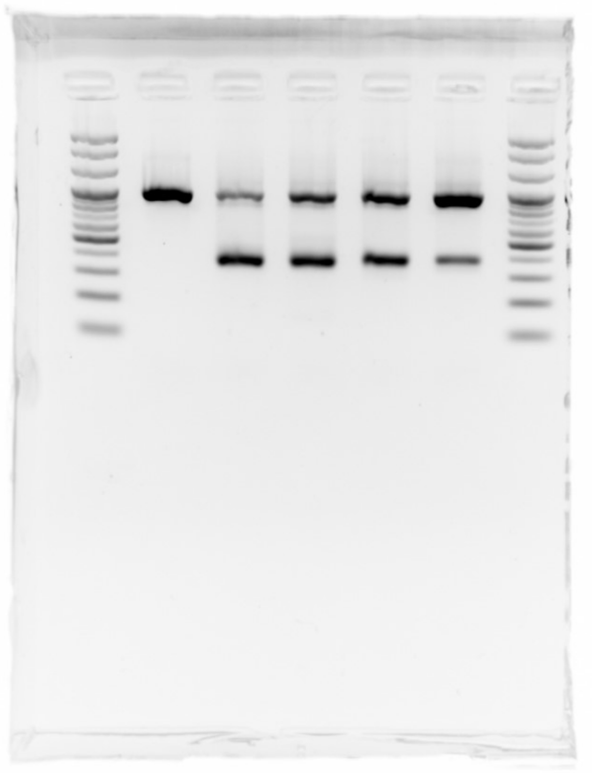

b. Unprocessed gel image for Figure 4b

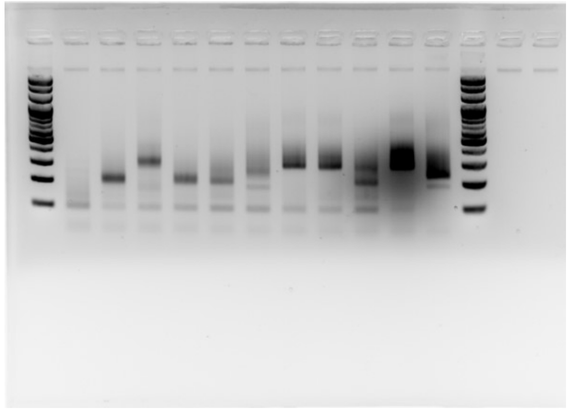

gRNA

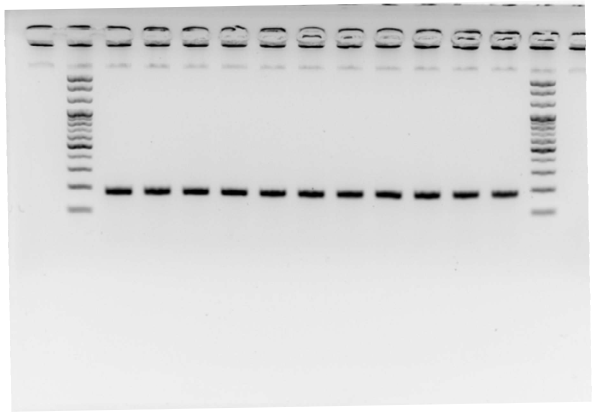

GAPDH

c. Unprocessed gel image for Figure 4d

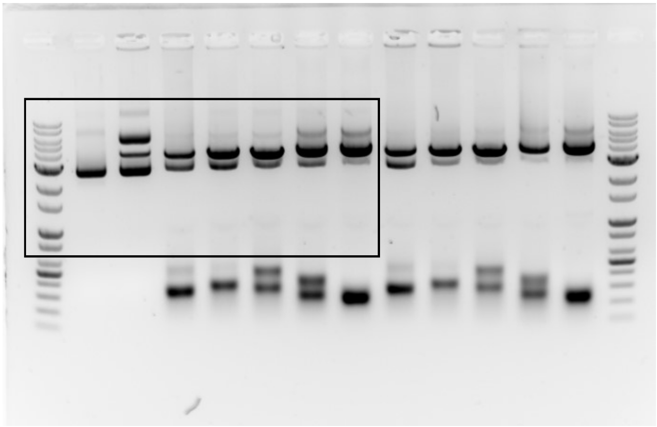

Supplement: Supplementary file 1 — Supplementary Figs. 1–10. [file 41587_2021_1009_MOESM1_ESM.pdf]
