# Supplementary material for: Efficient CRISPR editing with a hypercompact Cas12f1 and engineered guide RNAs delivered by adeno-associated virus
Source: Nat Biotechnol. 2021 Sep 2;40(1):94–102. doi: 10.1038/s41587-021-01009-z (PMC8763643; doi:10.1038/s41587-021-01009-z)
Supplement: Supplementary file 2 — Reporting Summary [file 41587_2021_1009_MOESM2_ESM.pdf]

nature research

Corresponding author(s): Yong-Sam Kim

Last updated by author(s): Jun 30, 2021

# Reporting Summary

Nature Research wishes to improve the reproducibility of the work that we publish. This form provides structure for consistency and transparency in reporting. For further information on Nature Research policies, see our Editorial Policies and the Editorial Policy Checklist.

## Statistics

For all statistical analyses, confirm that the following items are present in the figure legend, table legend, main text, or Methods section.

| n/a | Confirmed | |
|---|---|---|
| ☐ | ☒ | The exact sample size (*n*) for each experimental group/condition, given as a discrete number and unit of measurement |
| ☐ | ☒ | A statement on whether measurements were taken from distinct samples or whether the same sample was measured repeatedly |
| ☐ | ☒ | The statistical test(s) used AND whether they are one- or two-sided<br>*Only common tests should be described solely by name; describe more complex techniques in the Methods section.* |
| ☐ | ☒ | A description of all covariates tested |
| ☒ | ☐ | A description of any assumptions or corrections, such as tests of normality and adjustment for multiple comparisons |
| ☐ | ☒ | A full description of the statistical parameters including central tendency (e.g. means) or other basic estimates (e.g. regression coefficient) AND variation (e.g. standard deviation) or associated estimates of uncertainty (e.g. confidence intervals) |
| ☐ | ☒ | For null hypothesis testing, the test statistic (e.g. *F*, *t*, *r*) with confidence intervals, effect sizes, degrees of freedom and *P* value noted<br>*Give P values as exact values whenever suitable.* |
| ☒ | ☐ | For Bayesian analysis, information on the choice of priors and Markov chain Monte Carlo settings |
| ☒ | ☐ | For hierarchical and complex designs, identification of the appropriate level for tests and full reporting of outcomes |
| ☒ | ☐ | Estimates of effect sizes (e.g. Cohen's *d*, Pearson's *r*), indicating how they were calculated |

*Our web collection on statistics for biologists contains articles on many of the points above.*

## Software and code

Policy information about availability of computer code

| | |
|---|---|
| Data collection | Whole-genome sequencing data were collected using a fastqc (v0.11.8) for raw data QC, BWA-MEM (v0.7.17-r1188) for alignment, and Picard SortSam (v2.21.8) for alignment sorting installed in a DNBSEQ-T7 sequencing system (MGI). Deep sequencing data were collected using an iSeq Control software (v1.4.1.1700) installed in an iSeq 100 sequencer (Illumina). Quantitative RT-PCR data were collected using a QuantStudio Design & Analysis Software (v1.5.1). |
| Data analysis | SigmaPlot (v14.0), MAUND (available at https://github.com/ibs-cge/maund), Digenome-toolkit (available at https://github.com/chizksh/digenome-toolkit2). Excel (2016) |

For manuscripts utilizing custom algorithms or software that are central to the research but not yet described in published literature, software must be made available to editors and reviewers. We strongly encourage code deposition in a community repository (e.g. GitHub). See the Nature Research guidelines for submitting code & software for further information.

## Data

Policy information about availability of data

All manuscripts must include a data availability statement. This statement should provide the following information, where applicable:

- Accession codes, unique identifiers, or web links for publicly available datasets
- A list of figures that have associated raw data
- A description of any restrictions on data availability

Whole genome sequencing data were deposited at the NCBI Sequence Read Archive (SRA, http://www.ncbi.nlm.nih.gov/sra). Raw data are provided in the Source Data file.

# Field-specific reporting

Please select the one below that is the best fit for your research. If you are not sure, read the appropriate sections before making your selection.

☒ Life sciences ☐ Behavioural & social sciences ☐ Ecological, evolutionary & environmental sciences

For a reference copy of the document with all sections, see nature.com/documents/nr-reporting-summary-flat.pdf

# Life sciences study design

All studies must disclose on these points even when the disclosure is negative.

| | |
|---|---|
| Sample size | No statistical analyses were performed to predetermine sample sizes, but our study contains a large-scale validation experiment with a sample size of 88, which is the largest sample size among those reported until now for the efficiency assessment. |
| Data exclusions | No data were excluded. |
| Replication | All experiments were repeated at least three times. Critical experiments were confirmed for repeatability by 3 different testers. |
| Randomization | Mammalian cells used in this study were grown under identical conditions; no randomization was used |
| Blinding | Large-scale validations were performed by 3 different testers blinded to information with respect to the Cas type. |

# Reporting for specific materials, systems and methods

We require information from authors about some types of materials, experimental systems and methods used in many studies. Here, indicate whether each material, system or method listed is relevant to your study. If you are not sure if a list item applies to your research, read the appropriate section before selecting a response.

## Materials & experimental systems

| n/a | Involved in the study | |
|---|---|---|
| ☒ | ☐ | Antibodies |
| ☐ | ☒ | Eukaryotic cell lines |
| ☒ | ☐ | Palaeontology and archaeology |
| ☒ | ☐ | Animals and other organisms |
| ☒ | ☐ | Human research participants |
| ☒ | ☐ | Clinical data |
| ☒ | ☐ | Dual use research of concern |

## Methods

| n/a | Involved in the study | |
|---|---|---|
| ☒ | ☐ | ChIP-seq |
| ☒ | ☐ | Flow cytometry |
| ☒ | ☐ | MRI-based neuroimaging |

## Eukaryotic cell lines

Policy information about cell lines

| | |
|---|---|
| Cell line source(s) | HEK293T (Lenti-X 293T, Takara) |
| Authentication | Cells were authenticated by the supplier using STR analysis. |
| Mycoplasma contamination | Mycoplasma contamination was regularly tested and non-contaminated cells were only used in this study. |
| Commonly misidentified lines (See ICLAC register) | Not used. |
